# Unleashing potential: assessing Africa’s readiness for the data science revolution to impact health

**DOI:** 10.1038/s41467-026-71454-4

**Published:** 2026-04-13

**Authors:** Sumir Panji, Verena Ras, Judy Gichoya, Rolanda Julius, Suresh Maslamoney, Tshiamo Motshegwa, Abdoallah Sharaf, Michelle Skelton, Mahadia Tunga, Nicola Mulder

**Affiliations:** 1 Computational Biology Division, Department of Integrative Biomedical Sciences, Wellcome Discovery Research Platform for Infection, CIDRI-Africa, IDM, Faculty of Health Sciences, University of Cape Town, Cape Town, South Africa; 2 Emory University School of Medicine, Atlanta, GA USA; 3 National Research Foundation (NRF), Pretoria, South Africa; 4 SequAna Core Facility, Department of Biology, University of Konstanz, Konstanz, Germany; 5 Genetic Department, Faculty of Agriculture, Ain Shams University, Cairo, Egypt; 6 Department of Computer Science and Engineering, College of Information and Communication Technologies, University of Dar es Salaam, Dar es Salaam, Tanzania

**Keywords:** Computational platforms and environments, Translational research, Information technology

## Abstract

Data Science can revolutionize biomedical sciences, and ultimately, health. However, this relies on core elements, including computing infrastructure, data science skills, and well-curated contextually relevant data, which are limited in low- and middle-income countries. Many big data biomedical projects that have emerged over the last decade have been instrumental in development of datasets, computing infrastructure and skills; yet large disparities remain. Here, we review the data science development within Africa over recent years and identify key gaps and challenges that still hinder its implementation for health. We provide recommendations for establishing a sustainable data science ecosystem within Africa.

## Abstract (web summary)

This narrative review outlines Africa’s biomedical data science capacity, noting progress and remaining gaps. It recommends strengthening data and computing infrastructure and developing human expertise to better use data for improved health.

## Introduction

**Fig. 1:**
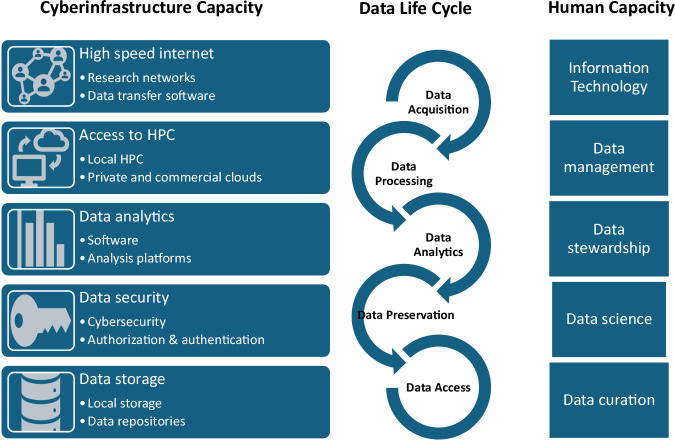
Needs across the data life cycle. Requirements for data science include infrastructure and human capacity for all aspects of the data life cycle (HPC is High Performance Computing).

Data are being generated at unprecedented rates in the biomedical sciences with concurrent development of new approaches for research and data usage. Traditional hypothesis-driven approaches investigating correlations between data types have been surpassed by the integration of large multimodal datasets and the application of data science techniques to generate new findings^1^. The availability of large volumes of health and biomedical data (big data) from multiple sources has enormous potential to facilitate novel discoveries and impact health care outcomes^2^ in a One Health approach. Despite this promise, big data poses a number of challenges, particularly in resource-limited settings where data science requires basic infrastructure, including access to storage and computing facilities, stable electricity supply, stable high-speed Internet, and multi-disciplinary skills. Implementation of data science projects encompasses all aspects of the data life cycle from data acquisition, curation, linkage, integration, analysis, visualization and interpretation to generate novel knowledge (Fig. 1). Additionally, data are often integrated with other datasets for further analysis, creating new derived data that should follow the later parts of the data lifecycle, including preservation and sharing. Implementing the data lifecycle requires specialist skills and secure infrastructure, which are typically limited in low- and middle-income countries (LMICs). However, even with modest infrastructure and access to data, data scientists can make an impact and be internationally competitive, making data science disciplines attractive for African scientists to pursue.

Recent investments in large-scale biomedical research in Africa have spurred the growth of data science by generating datasets relevant to African health problems, improved research infrastructure at African institutions, and in some cases, improved electricity supply and internet accessibility. An example is H3Africa, a $176 million investment from the National Institutes of Health (NIH) and Wellcome to investigate the genetic and environmental basis of communicable and non-communicable human diseases in Africa, while developing research capacity on the continent^3^. H3ABioNet, the bioinformatics network of H3Africa, has been instrumental in building bioinformatics capacity across Africa over more than a decade^4^. Using a diverse and extensive training program, H3ABioNet has built skills in all aspects of biological data, from systems administration for setting up information technology (IT) infrastructure for bioinformatics to data management, analysis and interpretation^5^ while considering local context and limitations^6,7^. H3ABioNet developed specific communities to meet local needs focusing on machine learning and computational and statistical skills, necessary for data scientists. New, upcoming initiatives, including the Africa Population Cohorts Consortium (https://ce-apcc.org/) and the network of Genomic Centers of Excellence, also have a capacity building focus. The Data Science Without Borders initiative led from Kenya, aims to strengthen data science systems in health, and develop platforms to facilitate the application of data science to health data.

More recently, the NIH invested in the establishment of the Harnessing Data Science for Health Discovery and Innovation in Africa (DS-I Africa) consortium (https://dsi-africa.org/), which aims to develop a network of African data scientists working to apply data science technologies to impact health. The consortium aims to extract maximum value out of limited datasets while integrating diverse dataset types to generate new insights into health. The consortium is building capacity by strengthening training capacity for short- and long-term needs and developing guidelines and policies, including assessment of international laws associated with health data through ethical, legal and social implications (ELSI) programs. These and future capacity building initiatives are critical in harnessing the promise of data science for better health in Africa across the dataset lifecycle in an ethical and responsible manner.

With increasing popularity of data science and the application opportunities it provides for biomedical research (even in LMICs), it is timely to reflect on the readiness of these countries to embrace the field. In this article, we review the current state of data science capacity in Africa, how this has progressed, and what needs to be done. We limit our scope to biomedical sciences in Africa focusing on the data lifecycle, infrastructure requirements, and human capacity. We describe the current data science landscape and identify gaps and opportunities framed in the context of recent developments. These findings informed recommendations for policy development and strategic planning toward building a pan continental data science ecosystem able to meet Africa’s health needs.

## Results and discussion

### Cyberinfrastructure to empower data science

While the data science lifecycle overall is dependent on a well-equipped IT infrastructure, here we will focus on the networking, data transfer, security, computing, and storage components (as well as human capital development) as essential components of a cyberinfrastructure^8^. While there are developments in various countries on elements of cyberinfrastructure, most are not guided by national, regional or continental frameworks, roadmaps, and implementation plans. South Africa, however, does follow the National Integrated Cyberinfrastructure System (NICIS: https://www.nicis.ac.za/) plan, and the Southern African Development Community (SADC) countries have developed and approved a Cyberinfrastructure Framework^9^ accompanied by relevant regional government ministries’ pronouncements towards its implementation. The development and implementation of these Cyberinfrastructure Frameworks can support data and handle intensive regional collaborative projects, including health.

### Networking: connecting the continent

Internet connectivity provides the backbone fabric of cyberinfrastructure. Significant progress has been made in the development of backbone telecommunications undersea cables on the eastern and western side of the continent; for example, through consortia for the EASSy (Eastern Africa Submarine System: https://www.submarinenetworks.com/en/systems/asia-europe-africa/eassy) and WACS (West Africa Cable System: https://www.submarinenetworks.com/en/systems/euro-africa/wacs/wacs-overview) projects, comprised mostly of African telecommunication companies (See: https://manypossibilities.net/african-undersea-cables/ for the locations of cable routes and connections). The cables connect Africa to other continents to carry and route international internet traffic. Extending connectivity inland incurs additional network costs to landlocked countries.

There has also been a lot of progress in network connectivity through the development of National Research and Education Network (NREN) and Regional Research and Education Networks (RRENs) (See: https://africaconnect3.net/wp-content/uploads/2024/02/AC3-Map-February-2024.jpg to view the African NREN connectivity network). A NREN is a specialized network provider dedicated to supporting domestic research and education community requirements. They are also essential for providing high-speed backbone network connectivity required for cyber-infrastructure platforms, including science gateways, data repositories, and research clouds. The World Bank report on “*The role and status of African National Research and Education Networks”*^10^ used the NREN Capability Maturity Model (CMM) to assess African NRENs; 9 African NRENs qualify as Level 6 (mature).

NREN infrastructure capacity in Africa has been progressively expanded through a series of EU partially-supported projects, in partnership with RRENs, NRENs, and capacity support partners. It is encouraging that the remainder of the funding is provided by African partners (https://africaconnect3.net/partners/). The series of projects include EUMEDCONNECT (2004–2015, see: https://eumedconnect3.net/), AfricaConnect (2011–2015, see: https://archive.geant.org/projects/africaconnect/ac1/Pages/Home.html) and AfricaConnect2 (2015–2019) that focused on commissioning high speed data networks, and improvement of bandwidth and reliable connection to other research and education communities globally (https://international-partnerships.ec.europa.eu/policies/programming/projects/africaconnect_en). African NRENs have varying governance, business, and sustainability models (See: The Case For NRENs resource https://casefornrens.org/ for some use cases from African NRENS). In general, a NREN can either be solely financed and driven by higher education and research institutions; or alternatively, it can be exclusively financed and driven by Government and embedded within a relevant Government Ministry; The Mozambique NREN (MoRENet) and Somalian SomaliREN are examples of this approach. However, the more common model is a hybrid of these, that is a dual financing model based on collaboration and contributions from member institutions, such as universities and research institutions, supported by Government with the NREN managed by the institutions. Zambian NREN (ZAMREN) and South African TENET are two examples of NRENs employing this model. There are advantages and disadvantages to the various models, but the latter mode does offer good avenues for sustainability, stability and a high degree of member institution engagement and ownership. On the downside, the model does incur coordination overheads.

The NRENs are looking to offer high performance computing facilities, advanced data services and support for open science. Avenues exist through the current ongoing phase 3 AfricaConnect3 project (https://africaconnect3.net/) that seeks to primarily focus on human capital development and promote digital transformation for research and education. The following NRENs already allow for running data-intensive applications and sharing of high-end computing and modeling assets: KENET (Kenya), TENET (South Africa), RENU (Uganda), and ZAMREM (Zambia)^11^. Significant progress has also been made in Algeria, Egypt, Kenya, Morocco, Senegal, Tunisia, South Africa, Uganda, and Zambia, connecting universities and research institutions to these assets. African NRENs and RRENs also run extensive human capacity building activities, for example, through the UbuntuConnect Series (https://ubuntunet.net/uc2024/).

### Facilitating data transfer

The ability to transport large datasets to and from High Performance Computing (HPC) systems is critical for data science. Data intensive, high-performance applications and workloads demand high data transfer rates, and traditional methods of data transfer such as HTTP and FTP are inadequate. The solution is typically a dedicated “science network”, as opposed to a general-purpose network, and network architectures explicitly designed for these purposes. A Science Demilitarized Zone (DMZ)^12^ is an example of such a network. Some NRENS in Africa, like SANREN, provide these advanced data services. At a software layer, specialized data transfer solutions and clients (such as Globus Online and Aspera, https://www.globus.org/)^13^, allow secure transfer and can resume transfer after interruptions in connectivity.

### Exploring Africa’s computational landscape for data science

High-performance computing systems provide the necessary power and scalability for handling big data. Traditionally, government organizations and NREN provided access to HPC or supercomputer systems. In South Africa, the national cyberinfrastructure provides the Center for High Performance Computing (https://www.chpc.ac.za/) while the Ilifu compute cluster (https://www.ilifu.ac.za/) was established by a consortium of South African universities, located at the University of Cape Town and operated by the Institute for Data Intensive Astronomy. The National Institute of Allergy and Infectious Diseases (NIAID) supports two bioinformatics centers of excellence, one in Mali and one in Uganda, which host reasonable computing facilities. The emergence of public cloud providers, such as Amazon Web Services (AWS), Google Cloud Platform (GCP), and Microsoft Azure has made HPC more accessible to researchers and scientists. However, many barriers to using commercial clouds, particularly for LMIC scientists, still exist, and as a result, there is a reliance on local computing facilities.

The HPC Discovery Survey (see: 10.31219/osf.io/6vkuh) received a total of 74 responses, 36 respondents completed the full survey. Based on the survey results, it is clear that access to computing infrastructure remains a challenge in many African countries. A further 18 respondents were consumers of computer resources but were unaware of the underlying infrastructure; therefore, they could only complete part of the survey. Responses represent 22 African countries with the highest set of responses (18%), from South Africa. The survey probed the location of the HPC resource used, if locally hosted or cloud based, and whether or not the HPC resource was available for collaboration. Of the 36 respondents, 28 (71.8%) used a locally hosted HPC, while 9 ( ~ 23%) used another institution’s HPC. None of the respondents had access to or used cloud based HPC resources. More than half of the respondents (58.5%) indicated that their HPC resource was available for collaboration, some (31.7%) were unsure, and others (9.8%) indicated that their HPC resources were unavailable for external collaboration. It is interesting to note that most of the computing environments were implemented between 2022 and 2024, so are reasonably new. Reported challenges include slow internet speeds, system downtime due to loadshedding (electricity supply outages), limited storage space (insufficient capacity and inefficient data management techniques), lack of bioinformatics skills for big data analysis, and lack of computer resources. For a more detailed description of the results, see: 10.31219/osf.io/6vkuh.

### Breaking barriers to accessing IT infrastructure

Health data requires controlled access to authorized individuals and data science tools need to be accessible and executable on the computing infrastructure, preferably through user-friendly interfaces. International solutions like Terra (https://terra.bio/) and Gen3 (https://gen3.org/), have been used for large-scale biomedical data initiatives. Several African cloud-based infrastructures, such as the African Open Science Platform (AOSP) and eLwazi Open Data Science Platform (ODSP) are being developed to remove or minimize the IT infrastructure barriers African data scientists encounter working with large datasets.

The eLwazi ODSP (http://www.elwazi.org) is being developed as part of the DS-I Africa consortium. eLwazi uses Terra and Gen3 to provide a flexible, scalable open data science platform for African researchers, supporting access to three of the main data science components: IT infrastructure, data, and data science tools. The platforms can be accessed via user workspaces and enable discovery of local and federated datasets, provide access to a suite of tools and workflows, and facilitate execution of analyses on selected computing infrastructures. The African Open Science Platform (https://aosp.org.za/), a broader continental initiative, aims to position African scientists at the cutting edge of data-intensive science by stimulating interactivity and creating opportunity through the development of efficiencies of scale, building critical mass through shared capacities, and amplifying impact through a commonality of purpose and voice. AOSP has established three regional nodes: the Egyptian National Authority for Remote Sensing and Space Sciences (NARSS) for the Northern African Node based in Egypt; the African Institute for Capacity Development (AICAD) for the East African Node based in Kenya, and the UbuntuNet Alliance (UA) for the Southern Africa Node based in Malawi (https://www.nrf.ac.za/the-african-open-science-platform-appoints-three-regional-nodes/). Ubuntunet Alliance, an alliance of Southern and East African NRENs, is developing community cloud infrastructure and capacity to support open science (https://africaconnect3.net/invitation-to-tender-for-cloud-infrastructure-equipment-ubuntunet-alliance/) for AOSP network stakeholders. There are other notable investments in HPC capacity, including in South Africa (1 PetaFlop system at CHPC in Cape Town), Morocco (3.15 Petaflop system hosted within the new African Supercomputing Center (ASCC) https://www.moroccoworldnews.com/2021/02/335518/moroccan-university-inaugurates-africas-most-powerful-supercomputer) and elsewhere. There is also a trajectory towards developing global research commons; for example, the Global Open Science Cloud (GOSC: https://codata.org/initiatives/decadal-programme2/global-open-science-cloud/) led by the International Science Council (ISC) Committee on Data (CODATA) initiative, which aims to foster cooperation, synergies, interoperability, and alignment between open science platforms globally and to use these platforms for joint action and collaboration.

### Maximizing Africa’s data assets for health

Data science for health advancements requires access to and integration of heterogeneous multidimensional data types, such as genomics, transcriptomics, proteomics, phenotypic, socio-demographic, mobility, imaging, climate modeling, infectious disease modeling, verbal autopsies, electronic health records, among others^14^. Maximizing the utility of this diverse data beyond the initial project can only be accomplished if properly managed through appropriate data acquisition strategies, use of community data models streamlined for interoperability and machine-readiness and preserved for long-term accessibility and global reuse. Efficient data acquisition strategies and robust data management plans play a pivotal role in using the data for advancing our understanding of health challenges and to help design appropriate intervention strategies^15^.

### Optimizing data management: storage solutions and repositories

Comprehensive policies for data management beyond a project’s life cycle at an Institutional level, and different levels of commitment for responsible data sharing are often lacking. A total of 229 data repositories in 41 African countries were identified in a 2020 study^16^ (10.5281/zenodo.3732274), the majority being Institutional repositories (https://kumu.io/access2perspectives/african-digital-research-repositories). There are notable gaps in the number of African hosted community digital repositories, with South Africa and Kenya having the most repositories registered within the Registry of Research Data Repositories within Africa (https://www.re3data.org/browse/by-country/), mainly provided by academic institutions in the case of South Africa. The CoreSealTrust is a formal process of assessing the trustworthiness of digital repositories based on a number of predefined metrics, operations, and processes. Of the 128 CoreSealTrust repositories, only 3 are located within Africa (https://amt.coretrustseal.org/certificates/). Few institutions within Africa have the funding, resources, and capacity to manage research data repositories, and most research funding for data generation projects encourage submission of research data to data repositories in the global North (such as the European Genome Phenome Archive (EGA)) for long term preservation and access, due to their robust technical infrastructure and experience in running such repositories. Uncertainty among research communities in Africa regarding best practices and need for comprehensive data management practices pose challenges. Data loss risks, particularly for stand-alone projects, data reuse opportunities, indigenous data sovereignty, and adherence to guiding principles are important considerations. The need for guidance and support in adopting best practices and incorporating principles, such as FAIR (Findable, Accessible, Interoperable, and Reusable; https://www.go-fair.org/fair-principles/) and CARE (Collective benefit, Authority to Control, Responsibility, Ethics)^17^ is recognized, especially for new researchers that should learn how to steward and manage increasing data volumes.

Advances in storage technologies, such as cloud-based solutions and high-performance computing infrastructures, have enabled cost-effective and scalable storage of data^18,19^. Cloud platforms designed for genomic data management offer policies and procedures to ensure data governance and security^18^. Africa has witnessed a burgeoning interest in genomics, leading to the establishment of various genomic data repositories that play a pivotal role in advancing scientific research and healthcare initiatives. These platforms have formal data ingestion processes, multiple tiers of data access with varying authentication and authorization requirements, and measures for data security and auditing^18^. The H3ABioNet African resources include a repository for genomic datasets and tools, promoting collaboration and knowledge-sharing across Africa^20^. It encourages researchers to contribute their genomic data to public resources such as the EGA, gnomAD, ClinVar, DECIPHER, and MatchMaker Exchange^21^, but provides African options as an alternative, such as the African Genome Variation Database for sharing aggregate frequency data. As more data are being generated and collected within Africa by various initiatives (such as H3Africa, DS-I Africa, the Wellcome Trust DELTAs program, Global Grand Challenges program and others), the quality of the data being collected, data models being used, data management and sharing of curated data and metadata for the wider scientific community is increasingly crucial. Fortunately, many research funding organizations now require data management plans to be submitted along with a research proposal.

### Deriving value: data capture, curation and analysis

The use of data acquisition systems that do not require a lot of IT infrastructure and technical overheads, such as the Research Electronic Data Capture (REDCap) project have gained popularity within low resource settings, where paper-based records are the prevalent method for clinical record keeping^22,23^. This has led to the creation of a community of data managers and users under REDCap Africa. Skills and capacity for active stewardship of data during collection, processing and sharing need to be developed and integrated alongside data science programs and initiatives. Data management courses, such as the virtual Research Data Management course hosted by H3ABioNet and eLwazi ODSP, have become popular, as evidenced by the number of enrolled participants, which has seen a steady increase from 104 applications in 2020 to 972 applications for the 2024 iteration (https://elwazi.org/trainings/26). Using standardized collection instruments and controlled vocabularies to map data to, facilitates interoperability and data harmonization with other datasets for reuse. African research groups and consortia such H3Africa, Data Science Without Borders (DWSB), and DS-I Africa are adopting, creating, and using standards such as the Observational Medical Outcomes Partnership (OMOP), Genomics Cohorts Knowledge Ontology (GECKO), or creating a minimum set of standards for data collection with data dictionaries for certain studies (https://zivahub.uct.ac.za/projects/H3ABioNet_H3Africa_Phenotype_Standards_Project/149305)^24,25^.

Curation involves assessing data quality, correcting errors and annotating metadata to enhance its usefulness for downstream^26,27^. Data curation practices include retrospective upgrading of historical datasets, present-oriented monitoring of ongoing data collections, and future-looking dissemination of data^28^. Provenance and data curation are important components for understanding data metrology^29^. Exploiting metadata can improve the process of data preparation by detecting data errors^29^. Organized and well-curated datasets are crucial for accurate, reproducible research and data reuse, underlining the importance of effective data management practices. However, most research projects in low resource settings do not have resources or skills for data curation, leaving a concerning gap in the creation of well-curated datasets.

The processing stage of converting raw data into meaningful biological insights requires significant computational power and specialized algorithms to handle massive datasets. This is particularly important due to the complexity and volume of health-related and genomics data and increasing use of large language models. Data analysis in Africa is hampered by access to sufficient computational infrastructure, as discussed earlier. In addition to increased access to high performance computing infrastructure, skills for creating and submitting jobs to utilize HPC resources and schedulers need to be developed as data science projects become more computationally intensive, often requiring access to GPUs for using machine learning methods. Programs such as the HPC Ecosystems Project run by the Southern African Development Community in preparation for the Square Kilometer Array, have successfully trained over 700 individuals in the use of HPCs^8^ and seeks to create an HPC community of practitioners. Some facilities such as the CHPC and Ilifu provide training videos and sessions for new users, but this training is usually provided to users at the point of access, due to its practical application. Commercial cloud vendors (such as AWS, GCP, and Azure) have their own training pathways and certification programs that provide technical knowledge on how to work in and deploy tools within their specific platforms. However, these vendor certification programs have a fee that is higher than what most African degree students can afford (in addition to paying to use their platform) and are more geared to industry professionals.

### Enhancing data access and sharing

Data deserts in many African countries reflect the digital divide, as many people do not have access to the services and systems used to generate the data, or those that are needed to train algorithms or to analyze data for decision-making. Efficient data sharing is vital in the health sector where collaboration across institutions and regions can advance knowledge and produce new insights. Technologies (such as high-speed networks and data-sharing platforms) have revolutionized the process, enabling seamless and secure transfer of large datasets across research institutions globally^30,31^. One of the restrictions to data reuse, however, is the lack of licensing of data. This is much harder to implement with health and genomics data studies that Institutional Review Boards approve and is usually under controlled access by a Data Access Committee (DAC). The creation and application of unambiguous data consent and reuse conditions, such as the Data Use Ontology (DUO) helps to identify the conditions under which controlled access data is allowed to be reused, making it easier for DACs to determine whether requests for controlled access data fall in line with the data reuse conditions based on informed consents. DUO has been used by the H3Africa Consortium to describe the access and reuse conditions of genomics datasets generated within that program (https://catalog.h3africa.org/). The ontology is also being used in the DS-I Africa Elwazi catalog.

The rapid growth in the field of data science has necessitated the development and implementation of legal frameworks to safeguard individual privacy and country-sensitive data. Some of the more commonly known data protection regulations are the General Data Protection Regulation (GDPR: https://gdpr-info.eu/) in Europe, the Health Insurance Portability and Accountability Act (HIPAA: https://www.cdc.gov/phlp/php/resources/health-insurance-portability-and-accountability-act-of-1996-hipaa.html) in the USA and the Protection of Personal Information Act (POPIA: https://popia.co.za/) in South Africa. Many other African countries (up to ~39 out of 55 countries), such as Kenya, Uganda, and Botswana have passed similar data protection legislation measures which hinders the sharing of sensitive, identifiable demographic, health and genomics data outside of a country’s borders. These regulations, among other things, define what categories of data are allowed to cross digital borders and the expected data security levels (See also: https://dataprotection.africa/ accessed 12/06/2024). Cross border sharing of digital health data records and other identifiable data (such as cohort and genomics data) is difficult to navigate in the absence of expert advice, as new data protection laws in many African countries come into effect (https://www.datalaw.africa/law/search_compare/).

### AI readiness for Africa’s health data

While investments in infrastructure, storage, and training have laid an important foundation^6,32^, the next frontier for biomedical data science in Africa lies in ensuring that data itself is structured, curated, and prepared for advanced applications such as artificial intelligence (AI) and machine learning. Reliable AI models require harmonized, well-annotated, and metadata-rich datasets to ensure reproducibility and generalizability^33,34^. Yet, much of Africa’s data remains fragmented, inconsistently labeled, or siloed across institutions, limiting its AI-readiness^35,36^. Current and past initiatives are addressing these gaps, H3ABioNet, for example, developed training and protocols for data management and curation along with recommended ontologies^6,37^, while efforts such as the OHDSI/OMOP model are being piloted to harmonize population health data^38^. Similarly, frameworks such as the FAIR and CARE principles provide critical guidance for metadata quality, interoperability, and ethical governance of sensitive genomic and health data^39,40^. A large challenge in developing and applying machine learning models is the availability of high-quality diverse datasets that can be used for model training and evaluation. Programs such as DS-I Africa, Data Science Without Borders, Grand Challenges, and others, seek to address this by creating a continental network of data scientists that produce high quality data for machine learning applications for improved health outcomes. Projects such as eLwazi host an annual data jamboree that brings together the data managers and stewards from the DS-I Africa projects to collectively work on mapping the data-to-data models and ontologies, data harmonization, and improving the FAIRness of the project data. This has enabled a community of data stewards and data managers to be built, similar to the REDCap Africa group. Despite these advances, a continent-wide strategy for AI readiness, including standardized ontologies, common data models, and routine quality control pipelines, remains absent. Building such standards, while also expanding expertise in data annotation and ontology development, will be crucial to fully leverage Africa’s growing computing capacity. Positioning AI readiness as a strategic priority, through coordinated policies, investment in standards, and embedding structured data practices into biomedical projects, will be essential to move from infrastructure readiness toward true data-driven discovery^41,42^.

### Developing a data-driven workforce

The demand for skilled professionals in data science, particularly in the biomedical sector, has escalated globally. In Africa, where the burden of communicable diseases remains high and healthcare systems often face significant challenges^3^, the role of data science is becoming vital. In the context of biomedical data science, the need for multi-disciplinary training is paramount^4^. Data scientists in this field must possess a diverse skill set that goes beyond traditional data analysis. They need to understand the nuances of data curation, including data cleaning, transformation, and integration, which are crucial for ensuring the quality and reliability of biomedical data^4^. Additionally, proficiency in data management is essential for handling large volumes of data generated in biomedical research and healthcare settings^4^. This includes knowledge of database management systems, data governance, and data security practices. Moreover, expertise in managing IT infrastructure is crucial for creating platforms that enable seamless access to cloud resources. This involves setting up and maintaining cloud-based environments that support data storage, processing, and analysis, as well as providing user-friendly interfaces for data access and collaboration. Traditional IT training is often not sufficient for providing the skills required for setting up IT systems capable of managing biomedical data and associated tools and for working with biomedical scientists.

### Contrasting capacity development approaches

**Fig. 2:**
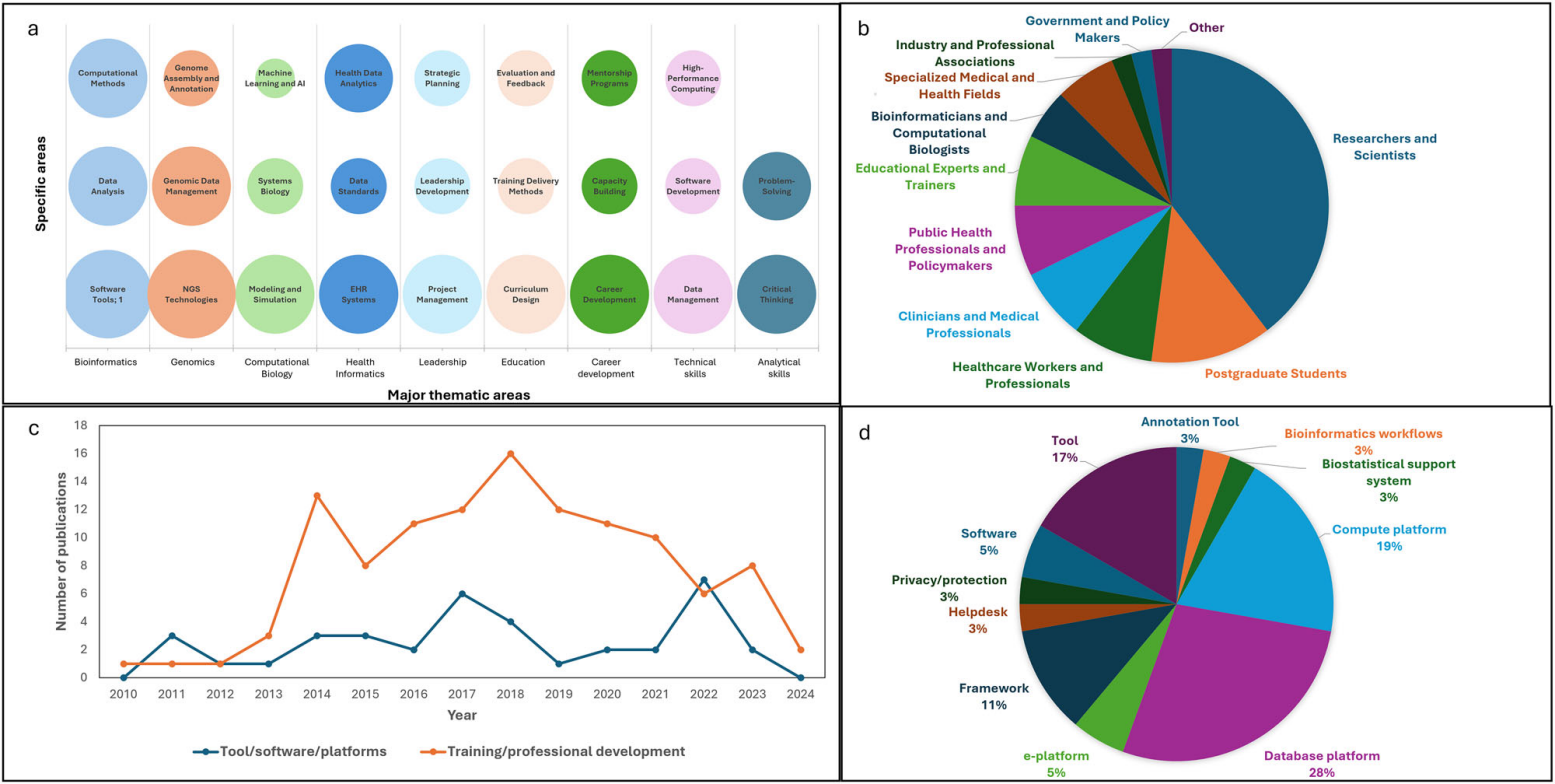
Summary of training information derived from published literature. **a** Bubble chart showing the distribution of major thematic and topic areas covered by published training initiatives (topics have been grouped into themes/topics and bubble sizes indicate the weighted frequency of each topic); **b** visual representation of audience distribution in training and capacity development programs; **c** number of publications over time focusing on capacity development in the form of training/professional development vs. tools/software (until March 12th 2024) and; **d** visual representation of major focus areas of development publications.

While our review provides an overview, it is important to note that it is not exhaustive. We identified several initiatives across Africa addressing the need for capacity building in biomedical data science, both formal and informal. Most published accounts of training efforts were related to bioinformatics, genomics, medical/health informatics, or biostatistics training as primary capacity-strengthening endeavors for biomedical data science (e.g.^4,32–36,38,43–48^; see Fig. 2a), and were mostly targeted towards researchers and professionals with little training within a policy space or teachers at primary or secondary school level (Fig. 2b). Only a few (<10/116) training publications described training on topics focused on developing the capacity for using cloud computing or other Information Technology or data management skills (e.g., see refs. ^37,39,45,49^). Although this could be due to the omission of computer science search terms from the original keyword list used during the literature search, it could also reflect a lack of uptake of these more professionalized skills within the biomedical community (see Fig. 2b).

Large pan-African or cross-boundary networks have made significant contributions to enhancing workforce readiness by building institutional and regional capacity. Programs such as H3ABioNet^4^ (www.h3abionet.org); AIMS (African Institute for Mathematical Sciences); African Centers of Excellence in Bioinformatics and Data Intensive Science^40^; the Collaborative African Genomics Network (CAfGEN)^46^; the African NGS Pathogen Surveillance Network^41^; the Sickle Cell Disease Genomics of Africa (SickleGenAfrica) Network^42^; and the Nigerian Bioinformatics and Genomics Network (NBGN)^50^, among others, have been successful in building capacity at an institutional level. These networks, particularly when offered coordinated funding for training, are able to focus on long-term aims and goals, the development of competency-based skills, and can share and distribute resources and expertise. This allows for the implementation of sustainable training and capacity-strengthening models, thereby increasing overall impact^5^. To further support capacity development, these networks have also supported the development of collaborative tools and infrastructure. For instance, over 30 publications retrieved during the literature screening focused on the development of collaborative tools, platforms, registries/databases, and open-source computing infrastructure, to support human capacity development (Fig. 2c), indicating a rise in the development of these tools since around 2017/2018 (Fig. 2d), shortly after some of these networks were formalized and funded. This demonstrates the impact of collaborative networks fueled by funding commitments, as these publications predominantly originated from nodes or affiliates of these larger networks. Other large Pan-African data science initiatives continue to foster communities of expertise such as the HPC project to capacitate Square Kilometer Array (SKA) countries that also focus on sustained human capital development as part of rolling out HPC equipment to centers within Africa (10.1145/3626203.3670537). While the roles of Research Software Engineers (RSEs) are explicitly recognized in high income countries, recognition of the role of RSEs within the African academic research environment is lagging without a clear career path as a traditional academic. There is currently a movement towards creating an African RSE community.

Networks like H3Africa and H3ABioNet have emphasized building institutional capacity, but they have also prioritized building individual capacity. For example, H3ABioNet offered distance- and competency-based training opportunities that have upskilled thousands of students, researchers, and IT professionals in bioinformatics and genomics analyses^5^. H3ABioNet has also provided support for the establishment of formal bioinformatics degree programs in some key regions across Africa, advancing both institutional and individual capacity^5^. Informal programs and networks, such as Deep Learning Indabas, Women in Data Science (WiDS), Data Science Africa, and The Carpentries, all offer further formal education, informal training, or mentorship opportunities aimed at upskilling and capacitating individuals within the data science space. Additionally, professional certifications for using public clouds, such as AWS, Azure, and Google Cloud (Supplementary Data 1), equip professionals with essential skills for leveraging cloud infrastructure, although their integration into formalized training within the biomedical sciences still appears relatively limited (Fig. 2a).

**Fig. 3:**
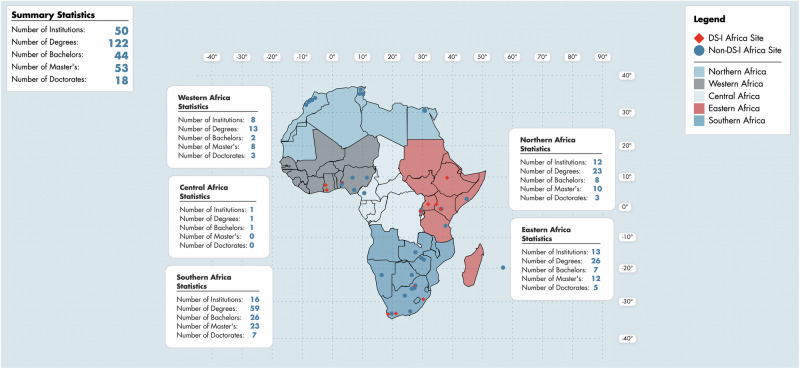
Distribution of data science degree programs in Africa, indicating where the DS-I Africa (Harnessing Data Science for Health Innovation in Africa) program will be initiating additional degree programs.

Analyses of Data Science related degree offerings at tertiary African institutions revealed that 122 programs are currently offered at 50 institutions. Southern Africa leads with 59 degree programs followed by Northern Africa with 23, Eastern Africa with 26, West Africa with 13 and only one in Central Africa. Thematic areas include Artificial Intelligence/Machine Learning; Bioinformatics/Genomics; Computational/Applied Mathematics; Computer Science; Data Science/Analytics; Information Science/Systems; Mathematics/Statistics; Software Engineering; Robotics, and others. The DS-I Africa Initiative added three additional institutions and 18 Degree offerings in 2022. The DS-I Africa Data Science programs offer more advanced domain-specific training related to public health, computational imaging, informatics, and omics. Three institutions will offer the full range of degrees from Bachelor of Science to PhD, while seven institutions will offer PhD programs (See the regional distribution of degree programs in Fig. 3 and more specific details in supplementary Data 2). The DS-I Africa Data Science Training programs supported by the Fogarty International Center endeavor to produce 157 Masters and 20 doctoral degree graduates and are expected to support 16 post-doctoral researchers and 51 faculty members for mentorship.

### Building on past successes: strategies for addressing gaps and challenges

Despite recent progress in building capacity in biomedical data science, significant gaps and needs persist. Few funded initiatives in Africa focus on building a critical mass for biomedical data science, leaving a major gap, especially with the conclusion of funding for initiatives like H3Africa and soon DS-I Africa. Current funding trends still indicate a flow of funding from the Global North to the Global South, primarily from major funders like the US National Institutes of Health (NIH) and the UK’s Wellcome Trust, while investments from African governments are limited. However, it is encouraging to note increasing funding from local governments for national or regional initiatives in recent years^51,52^. While it would be impossible to provide an exhaustive analysis of all of the gaps and challenges presented across the literature, major themes are summarized below:

- Few genomics facilities, insufficient numbers of trained personnel, and a lack of sustainable models for developing infrastructure to support advanced training and research remain major challenges across many LMICs.
- The adoption of digital health initiatives is often hindered by equity issues and low health and digital literacy.
- Many countries lack robust legislation and regulatory bodies for biomedical research.
- Many countries do not have adequate regulatory systems for educational certifications.
- Coordination difficulties and logistical challenges hinder the forming of effective South-South collaborations/partnerships.
- North-South collaborations often create power imbalances, maintaining reliance on external funding sources, and limiting autonomous research capacity.
- The scarcity of advanced biomedical data science postgraduate programs, limited leadership training and lack of advanced mentoring opportunities contribute to skill shortages.
- Gaps in the implementation of science training include the lack of scalable degree programs and locally relevant training models in LMICs.
- Developing a competent workforce and sustainable training models is hindered by the limited availability of skilled professionals, system weaknesses and a lack of advanced training opportunities in specialized fields like precision medicine and bioinformatics.
- A lack of interdisciplinary training, guidance on non-traditional roles, and limited pathways for skill enhancement beyond conventional research and health careers, constrain career development, leading to brain drain.
- Political instability and resource diversion in conflict settings compromise capacity-building efforts in affected regions.
- Complex disease burdens, skill shortages, and resource constraints pose significant challenges to mentoring efforts and impede institutional development in global health research.

Some recommendations to address these gaps and barriers are proposed in the final section, but one of these is an *African Biomedical Competency Framework* tailored to the African context, coupled with an *African Data Science Skills and Competence Ontology* to provide a standardized framework for skills development. This can help align training programs with industry needs and ensure that data scientists are equipped with the right knowledge and skills. Furthermore, harmonizing health data science frameworks across Africa can help standardize training and professional development programs, making it easier for data scientists to collaborate and share best practices. The Training Working Group within the DS-I Africa Initiative has already embarked on establishing a biomedical data science competency framework, geared at capacitating the various personas that exist within the consortium in biomedical data science. The framework draws on previous competency efforts by the International Society for Computational Biology (ISCB)^53,54^, Edison^55^ and IBM^56^, and has already identified ~20 core competencies to form the base of the framework. This framework will apply to several personas, ranging from data science users to data scientists and data engineers, which, after lengthy stakeholder consultation, were identified as already existing and needing development within this consortium.

The rise of networks, consortia, and cross-boundary initiatives emphasizes the need for more adaptive education models, including blended and distance-based learning models, which can reach a wider audience and sustain training efforts for longer periods^5^. There also seems to be a growing emphasis on mentorship and professional development programs^48,57–60^. Adopting decentralized training models, such as The Carpentries and the “Multiple-Delivery-Mode Learning Model” spearheaded by H3ABioNet^44^, can enhance the accessibility and scalability of training programs^38^. These models empower local communities to take ownership of their learning and development, leading to more sustainable capacity-building efforts^5^.

### Charting a path for advancement of African data science innovation and impact

This review has described the key requirements of a data science ecosystem for deriving value from biomedical data, including IT and computational infrastructure, access to and management of data, and availability of a broad range of data-related skills. While challenges exist in all components of this ecosystem globally, these are far more acute in LMICs, notably across the African continent. Data science infrastructure in Africa has improved over the past decade, largely through internationally funded consortia generating large datasets. Taking advantage of these opportunities, African scientists have been resourceful in improving their capacity to compete on the international stage in biomedical data science; however, gaps and challenges remain. One of the overarching themes across the 3 components discussed is the lack of investment from governments or local institutions in areas most needed to accelerate African data science, such as computing facilities, internet and electricity, specialized training and professional development, data access and data infrastructure. Aside from some exceptions, there is a general lack of road maps at all levels, including institutional, national, regional, and continental, to drive and guide development, implementation, and sustainability of cyberinfrastructure, research infrastructure, and open data platforms, and to promote ecosystems around their efficient use. Retention of African data scientists in the field of health data science is also a critical challenge. Investment in permanent positions for data scientists with competitive salaries and enticing projects would help to curb the brain drain.

The need for open, free, and universally implementable protocols for data reuse, including authentication and authorization procedures, is necessary. Licensing for data reuse and mapping to the Data Use Ontology, would facilitate recognition of original data providers and tracking the impact of datasets through citations. However, other significant barriers to data sharing and reuse remain. In 2019, over half of African countries lacked laws governing data sharing or protection or open data policies. To address this, the African Union adopted a draft data policy framework in early 2022, providing a non-binding blueprint for establishing robust data governance mechanisms. This framework aims to support harmonized regional data ecosystems through interoperability and cross-border data sharing. Since then, more countries have adopted data protection laws, and scientists need to navigate the complexities of lawful data sharing. In addition to policy frameworks, the role of communities like REDCap Africa is crucial. They provide platforms for data managers and offer research data management (RDM) training, addressing the high demand for such skills. These efforts are pivotal for building capacity in data science and ensuring the effective use and sharing of health data across the continent. Much of this use in the future is likely to be through AI technologies, increasing the imperative for AI-ready datasets.

### Recommendations for the future

Based on the outcome of our review, we recommend that all stakeholders, including funders, government, regional government groupings (e.g., SADC), industry, higher education, and research communities work together to address the remaining gaps and challenges that hinder data science applications in Africa. Here, we list a few recommendations that could have an enormous impact on the ability of African scientists to embrace data science techniques for biomedical research and innovation.

### IT and Infrastructure

1. Governments and institutions need to improve access to high-speed networking and internet. This requires stable electricity, government contributions and engagement with NRENs;
2. NRENs should be capacitated to provide advanced services for research, working with higher education institutions on dedicated research networks, and facilitate existing instruments for implementation and sustainability;
3. Computing facilities need to improve access to existing HPCs through embedded domain experts to work with the data science community. They should facilitate access for public health institutes and small to medium enterprises (SMEs);
4. Governments should invest in national data centers for computer and secure data storage, with transparent data governance policies. These should be implemented through digital transformation strategies and made accessible to researchers at subsidized costs. It is also important to develop skills in how to use such facilities, for example in HPC scheduling, and parallelizing code, and in how to access cloud computing;
5. It is important to invest in an African Research/Open Science Cloud (AOSC) to supplement national data centers. This may be inter-institutional or inter-governmental, depending on which countries have national HPCs.

### Data management

6) Institutions need to increase awareness of the importance of research data management (including metadata) and FAIR, e.g. through including these in university curricula, working with international FAIR communities, and through the development of institutional data management policies and data repositories;
7) Development of African trusted data repositories should be supported to increase accessibility of African data;
8) African-relevant data assets should be developed so that cleaned curated datasets are findable and machine-ready;

### Human capacity development

9) Recognition of professional careers in data management and stewardship should be encouraged and facilitated by creating relevant professional bodies. Existing professional bodies should add recognition for data science skills where relevant;
10) Specialized training for IT specialists should be developed to enable them to support data science requirements;
11) A minimum skill set for biomedical data scientists should be defined;
12) Training initiatives should design and implement a biomedical data science competency framework for data scientists and data science trainers to drive the quality and standardization of data science training programs and professional development;
13) The availability of expedited accredited formal education and informal training in biomedical data science needs to be increased. There is a need for more streamlined processes for introducing new degree programs, as accrediting new programs takes too long, during which time the field has changed;
14) Postgraduate biomedical students should be encouraged to take data science courses and grassroots communities in data science should be encouraged and strengthened;
15) Career paths with competitive salaries for data science workforce should be provided; for example, through partnership between academic institutions, government and industry;
16) There is a need to develop virtual connected African or national Data Science Institutes to provide a network of skills and encourage collaboration.

We believe that implementing some or all of these recommendations will go a long way towards improving Africa’s readiness for data science in the biomedical field.

In conclusion, Africa faces several unique challenges in strengthening data science capacity in the biomedical sector. However, successes, such as the contribution of African scientists to pandemic response efforts, demonstrate the potential impact of local expertise^61^. By nurturing talent within the continent and fostering trust in African data scientists, brain drain can be mitigated, enabling retention of critical skills. Promoting collaborations among African countries can also facilitate knowledge and resource sharing, leading to more effective and sustainable capacity building efforts. Looking ahead, it is imperative to increase the number of African data scientists over the next decade. By leveraging existing initiatives, identifying and addressing gaps, and fostering collaboration, Africa can cultivate a skilled workforce equipped to drive innovation and improve health outcomes on the continent.

## Methods

This paper adopts a narrative review approach to examine capacity development for data science in Africa across three interlinked themes: (1) IT and infrastructure, (2) data management, and (3) human capacity. A narrative design was chosen because the field is rapidly evolving, evidence is distributed across peer-reviewed and gray literature, and initiatives often document progress in diverse formats. Our goal was to synthesize and contextualize insights, not to conduct a systematic evidence audit.

To explore data stewardship practices, we drew on policies, frameworks, program documentation, and published accounts describing research data management (RDM) in African contexts. This included national and institutional data policies, repository guidelines, governance frameworks, and FAIR/open science strategies. Gray literature such as white papers, agency reports, and project websites was especially valuable where peer-reviewed evidence was sparse. We focused on themes such as governance and sovereignty, repository use, metadata standards, curation workflows, and the role of dedicated data stewards. Materials were compared across sectors to identify common enablers, persistent gaps, and examples of emerging best practice.

Evidence on computer, storage, networking, and cloud resources was sourced from facility reports, NREN publications, institutional websites, and peer-reviewed case studies. These were supplemented with insights from the “HPC Resource in Africa—Discovery Survey” (Supplementary Information), developed by the H3ABioNet consortium (https://h3abionet.org/) in partnership with the African BioGenome Project (https://africanbiogenome.org/), DS-I Africa (https://dsi-africa.org/), eLwazi (https://elwazi.org/) and the HPC (High Performance Computing) Ecosystems Project under the National integrated CyberInfrastructure System (https://www.nicis.ac.za/). The original survey was targeted at systems administrators and other custodians of infrastructure; some responses were also received from general staff. For this review, we used the aggregated survey results only descriptively, to provide context where published sources were limited. We did not attempt formal statistical analysis or generalization. Within this theme we examined: facility scale and characteristics, access policies, support and enablement mechanisms, sustainability models, and barriers such as latency, cost recovery, or limited technical expertise. Importantly, infrastructure is treated here as an enabler of human capacity, rather than an endpoint.

Human capacity development was explored through evidence on training, mentorship, and career pathways. Sources included peer-reviewed literature, descriptions of degree and certificate programs, professional development initiatives, and stakeholder input from networks involved in data science and bioinformatics across Africa. Gray literature such as course portals, institutional announcements, and program websites, was reviewed to capture recent developments not yet represented in academic publishing.

We characterized:

- Target audiences (students, analysts, engineers, educators, policy specialists).
- Training modalities (formal degree programs, non-accredited courses, cloud certifications).
- Mentorship ecosystems and communities of practice.
- Career and retention mechanisms, including incentives and diaspora engagement.

Particular attention was given to progression pathways (from foundational to advanced competencies) and to barriers such as brain drain, sustainability of training, and limited mentorship capacity.

Findings from each thematic area were integrated thematically rather than through quantitative aggregation. As a narrative review, this work is not exhaustive and is subject to publication and availability biases, especially in fast-moving initiatives documented outside traditional publishing channels. The HPC Discovery Survey was designed for infrastructure custodians, not end-users, and is used here only for contextual illustration. A continent-wide, user-focused survey of training and infrastructure needs would provide valuable complementary evidence but falls outside the scope of this review.

## Supplementary information

Supplementary Information

Description of Additional Supplementary Files

Supplementary Data 1

Supplementary Data 2

## Data Availability

Data for this review were sourced from websites, the literature, through PubMed, and the results of the HPC survey available at: 10.31219/osf.io/6vkuh. Data used to create figures are available in the Supplementary Information.

## References

[1] Navarro, F. C. P. et al. Genomics and data science: An application within an umbrella. *Genome Biol.* **20**, 109 (2019). 31142351 10.1186/s13059-019-1724-1 PMC6540394

[2] Hassan, M. et al. Innovations in genomics and big data analytics for personalized medicine and health care: A review. *Int. J. Mol. Sci.* **23**, 4645 (2022). 35563034 10.3390/ijms23094645 PMC9104788

[3] H3Africa Consortium et al. Research capacity. Enabling the genomic revolution in Africa. *Sci. (N. Y.)* **344**, 1346–1348 (2014). 10.1126/science.1251546 PMC4138491 24948725

[4] Mulder, N. J. et al. H3ABioNet Consortium. H3ABioNet, a sustainable pan-African bioinformatics network for human heredity and health in Africa. *Genome Res* **26**, 271–277 (2016). 26627985 10.1101/gr.196295.115 PMC4728379

[5] Aron, S. et al. The development of a sustainable bioinformatics training environment within the H3Africa Bioinformatics Network (H3ABioNet). *Front. Educ*. **6**, 10.3389/feduc.2021.725702 (2021).

[6] Parker, Z. et al. Building infrastructure for African human genomic data management. *Data Sci. J.* **18**, 47 (2019).

[7] Mulder, N. J. et al. H3ABioNet Consortium (as members of the H3Africa Consortium). Development of bioinformatics infrastructure for genomics research. *Global Heart* **12**, 91–98 (2017). 10.1016/j.gheart.2017.01.005 PMC5582980 28302555

[8] Johnston, B., Timm, L., Macleod, D. & Poole, J. Ten years of the HPC Ecosystems Project - Transforming HPC in Africa for the past decade. In *PEARC ‘24: Practice and Experience in Advanced Research Computing 2024: Human Powered Computing*, Article 35, 1–8, 10.1145/3626203.367053 (2024).

[9] Motshegwa, T., Wright, C., Sithole, H., Ngolwe, C. & Morgan, A. Developing a cyber-infrastructure for enhancing regional collaboration on education, research, science, technology and innovation. In *2018 IST-Africa Week Conference (IST-Africa)*, 1–9 (IEEE, 2018). 10.23919/ISTAFRICA.2018.8417349.

[10] Foley, M. The Role and Status of National Research and Education Networks in Africa Open Knowledge Repository. 10.1596/26258 (2016).

[11] Academy of Sciences of South Africa, African Open Science Platform Part 1: Landscape Study 2019, https://research.assaf.org.za/items/b07d1693-7279-4115-89dd-f4be7a145587

[12] Crichigno, J., Bou-Harb, E. & Ghani, N. A comprehensive tutorial on Science DMZ. *IEEE Commun. Surv. Tutor.* **21**, 2041–2078 (2019).

[13] Foster, I. Globus Online: Accelerating and democratizing science through cloud-based services. *IEEE Internet Comput.* **15**, 70–73 (2011).

[14] Munyaradzi, M. New funding set to transform data science research and innovation: Essential building blocks for the continent’s development. *Nature* (News Feature). 10.1038/d44148-021-00111-3 (2021).

[15] Wenham, C. et al. Measuring health science research and development in Africa: Mapping the available data. *Health Res. Policy Syst.* **19**, 142 (2021). 34895277 10.1186/s12961-021-00778-y PMC8665309

[16] Bezuidenhout, L., Havemann, J., Kitchen, S., De Mutiis, A., & Owango, J., African digital research repositories: Mapping the landscape. Curated by Zenodo 10.5281/zenodo.3732171 (2020).

[17] Carroll, S. R. et al. The CARE principles for indigenous data governance. *Data Sci. J.* **19**, 43 (2020).

[18] Dahlquist, J. M., Nelson, S. C. & Fullerton, S. M. Cloud-based biomedical data storage and analysis for genomic research: Landscape analysis of data governance in emerging NIH-supported platforms. *HGG Adv.* **4**, 100196 (2023). 37181330 10.1016/j.xhgg.2023.100196 PMC10173774

[19] Hofmann, P., Cabrera, J. A., Krieg, E., Bassoli, R. & Fitzek, F. H. P. DNA-storage in future communication networks. *IEEE Commun. Mag.* **61**, 178–183 (2023).

[20] Schultz, B. et al. on behalf of H3Africa Ethics and Community Engagement Working Group. Webinar report: Stakeholder perspectives on informed consent for the use of genomic data by commercial entities. *J. Med. Ethics* **50**, 57–61 (2023). 36941048 10.1136/jme-2022-108650 PMC10804035

[21] Carey, M. E. et al. Unlocking the potential of genomic data to inform typhoid fever control policy: Supportive resources for genomic data generation, analysis, and visualization. *Open Forum Infect. Dis.* **10**, S38–S46 (2023). 37274533 10.1093/ofid/ofad044 PMC10236510

[22] Maré, I. A. et al. Electronic data capture system (REDCap) for health care research and training in a resource-constrained environment: Technology adoption case study. *JMIR Med. Inf.* **10**, e33402 (2022). 10.2196/33402 PMC9472062 36040763

[23] Odukoya, O. et al. Application of the research electronic data capture (REDCap) system in a low- and middle-income country– experiences, lessons, and challenges. *Health Technol. (Berl.)* **11**, 1297–1304 (2021). 35251887 10.1007/s12553-021-00600-3 PMC8896572

[24] Kiwuwa-Muyingo, S., Todd, J., Bhattacharjee, T., Taylor, A. & Greenfield, J. Enabling data sharing and utilization for African population health data using OHDSI tools with an OMOP-common data model. *Front. Public Health* **11**, 1116682 (2023). 37361151 10.3389/fpubh.2023.1116682 PMC10287979

[25] Lawson, J. et al. The data use ontology to streamline responsible access to human biomedical datasets. *Cell Genomics* **1**, 100028 (2021). 34820659 10.1016/j.xgen.2021.100028 PMC8591903

[26] Parmiggiani, E., Amagyei, N. & Kollerud, S. K. S. Data curation as anticipatory generification in data infrastructure. *Eur. J. Inform. Syst.* **33**, 748–767 (2023).

[27] Cheney, J., Chapman, A., Davidson, J., & Forbes, A. B. Data provenance, curation and quality in metrology, pp. 167-187). In *Advanced Mathematical and Computational Tools in Metrology and Testing* XII 10.1142/9789811242380_0009 (2022).

[28] Visengeriyeva, L. & Abedjan, Z. Anatomy of metadata for data curation. *J. Data Inf. Qual.* **12**, 16–30 (2020).

[29] Krishnam, N. et al. multi-disciplinary data quality curation framework for discrete and continuous IoT data, pp. 376-382. In *2022 11th International Conference on System Modeling & Advancement in Research Trends (SMART)*, Moradabad, India. 10.1109/SMART55829.2022.10047227 (2022).

[30] Alvarellos, M. et al. Democratizing clinical-genomic data: How federated platforms can promote benefits sharing in genomics. *Front. Genet.* **13**, 1045450 (2023). 36704354 10.3389/fgene.2022.1045450 PMC9871385

[31] Kumuthini, J. et al. Genomics data sharing. In *Genomic Data Sharing Case Studies, Challenges, and Opportunities for Precision Medicine* (ed. Mccormick, J. & Pathak, J.) (Academic Press, 2023). 10.1016/B978-0-12-819803-2.00003-1.

[32] Guerfali, F. Z., Laouini, D., Boudabous, A. & Tekaia, F. Designing and running an advanced bioinformatics and genome analyses course in Tunisia. *PLoS Comput. Biol.* **15**, e1006373 (2019). 30689625 10.1371/journal.pcbi.1006373 PMC6349305

[33] Onywera, H. et al. Boosting pathogen genomics and bioinformatics workforce in Africa. *Lancet Infect. Dis.* **24**, e106–e112 (2024). 37778362 10.1016/S1473-3099(23)00394-8

[34] Foster, G.& Nash, J. Introducing health informatics as an elective module in an information systems honours degree: Experiences from Rhodes University. In *ICT Education. SACLA 2016. Communications in Computer and Information Science* (ed. Gruner, S.) Vol. 642 (Springer, Cham., 2016). 10.1007/978-3-319-47680-3_12.

[35] Moodley, D., Pillay, A. W., & Seebregts, C. S. Establishing a health informatics research lab in South Africa. In *Proc. NEMISA 2018 Digital Re-imagination Colloquium: Preparing South Africa for a Digital Future through e-Skills*, 16–24 (National ElectronicMedia Institute of South Africa, 2018). https://uir.unisa.ac.za/handle/10500/25615.

[36] Nyangena, J. et al. Developing harmonized benchmarks for the Master of Science in Health Informatics for the East African region. *Stud. Health Technol. Inform.* **290**, 907–911 (2022). 35673150 10.3233/SHTI220211

[37] Maojo, V. et al. Biomedical informatics publications: A global perspective: part II: journals. *Methods Inf. Med.* **51**, 131–137 (2012). 22311187 10.3414/ME11-01-0061

[38] Ras, V. et al. Using a multiple-delivery-mode training approach to develop local capacity and infrastructure for advanced bioinformatics in Africa. *PLOS Comput. Biol.* **17**, e1008640 (2021). 10.1371/journal.pcbi.1008640 PMC7906323 33630830

[39] Asana, L. et al. Using advanced information and communication technologies to advance oncology education in Africa. *Ecancermedicalscience* **15**, 1211 (2021). 10.3332/ecancer.2021.1211 PMC8057784 33912236

[40] Giovanni, M. Y. et al. African centers of excellence in bioinformatics and data intensive science: Building capacity for enhancing data intensive infectious diseases research in Africa. *J. Infect. Dis. Microbiol.* **1**, 006 (2023). 37987019 10.37191/mapsci-jidm-1(2)-006 PMC10658664

[41] Christoffels, A. et al. A pan-African pathogen genomics data sharing platform to support disease outbreaks. *Nat. Med* **29**, 1052–1055 (2023). 37161068 10.1038/s41591-023-02266-y PMC12181013

[42] Anie, K. A. et al. & SickleGenAfrica Network. Sickle Cell Disease Genomics of Africa (SickleGenAfrica) Network: Ethical framework and initial qualitative findings from community engagement in Ghana, Nigeria and Tanzania. *BMJ Open* **11**, e048208. (2021). 10.1136/bmjopen-2020-048208 PMC8311318 34301659

[43] Isak, S. Increasing bioinformatics in Third World countries: Studies of *S. digitata* and *P. polymyxa* to further bioinformatics in East Africa (Dissertation). Retrieved from https://urn.kb.se/resolve?urn=urn:nbn:se:uu:diva-293636 (2016).

[44] Gurwitz, K. T. et al. Designing a course model for distance-based online bioinformatics training in Africa: The H3ABioNet experience. *PLOS Comput. Biol.* **13**, e1005715 (2017). 28981516 10.1371/journal.pcbi.1005715 PMC5628786

[45] Ahmed, A. E., et al. Organizing and running bioinformatics hackathons within Africa: The H3ABioNet cloud computing experience [version 2; peer review: 2 approved; 1 approved with reservations]. *AAS Open Res.* **1**, 10.12688/aasopenres.12847.1 (2019). 10.12688/aasopenres.12847.1 PMC7194140 32382696

[46] Mboowa, G. et al. The Collaborative African Genomics Network (CAfGEN): Applying genomic technologies to probe host factors important to the progression of HIV and HIV-tuberculosis infection in sub-Saharan Africa [version 2; peer review: 2 approved]. *AAS Open Res*. **1**, 3 (2018). 30714022 10.12688/aasopenres.12832.1 PMC6358002

[47] Msomi, N., Mlisana, K., de Oliveira, T., & Network for Genomic Surveillance in South Africa writing group. A genomics network established to respond rapidly to public health threats in South Africa. *Lancet Microbe* **1**, e229–e230. (2020). 10.1016/S2666-5247(20)30116-6 PMC7434425 32838349

[48] Abrudan, M., et al. & NIHR global health research unit on genomic surveillance of antimicrobial resistance. train-the-trainer as an effective approach to building global networks of experts in genomic surveillance of antimicrobial resistance (AMR). *Clin. Infect. Dis*. **73**, S283–S289 (2021). 10.1093/cid/ciab770 PMC8634536 34850831

[49] CRIT Care Asia et al. Leveraging a cloud-based critical care registry for COVID-19 pandemic surveillance and research in low- and middle-income countries. *JMIR Public Health Surveill.* **6**, e21939 (2020). 10.2196/21939 PMC7717923 33147162

[50] Fatumo, S. et al. The Nigerian Bioinformatics and Genomics Network (NBGN): A collaborative platform to advance bioinformatics and genomics in Nigeria. *Glob. Health Epidemiol. Genom*. **5**, e3 (2020). 10.1017/gheg.2020.3 PMC7372179 32742665

[51] Gwagwa, A., Kraemer-Mbula, E., Rizk, N., Rutenberg, I. & De Beer, J. Artificial intelligence (AI) deployments in Africa: Benefits, challenges and policy dimensions. *AJIC* **26**, 1–23 (2020).

[52] Adebamowo, C. A. et al. The promise of data science for health research in Africa. *Nat. Commun.* **14**, 6084 (2023). 37770478 10.1038/s41467-023-41809-2 PMC10539491

[53] Mulder, N. et al. The development and application of bioinformatics core competencies to improve bioinformatics training and education. *PLoS Comput. Biol.* **14**, e1005772 (2018). 29390004 10.1371/journal.pcbi.1005772 PMC5794068

[54] Brooksbank, C. et al. The ISCB competency framework v. 3: A revised and extended standard for bioinformatics education and training. *Bioinform. Adv.* **4**, vbae166 (2024). 39678208 10.1093/bioadv/vbae166 PMC11646570

[55] Demchenko, Y. et al. EDISON Data Science Framework: Part 1. Data Science Competence Framework (CF-DS) Release 4. Zenodo, Dec. 10.5281/zenodo.7506444 (2022).

[56] IBM Corporation. The data science skills competency model: A blueprint for the growing data scientist profession. IBM Corporation. Retrieved from https://www.scribd.com/document/468977131/55029955-usen-00-55029955USEN (2020).

[57] Phipps, W. et al. Peer mentoring at the Uganda Cancer Institute: A novel model for career development of clinician-scientists in resource-limited settings. *J. Glob. Oncol.* **4**, 1–11 (2018). 10.1200/JGO.17.00134 PMC6223430 30241258

[58] Pillai, G. et al. The Next Generation Scientist program: Capacity-building for future scientific leaders in low- and middle-income countries. *BMC Med. Educ.* **18**, 233 (2018). 30305069 10.1186/s12909-018-1331-y PMC6180641

[59] Ruiz-Alzola, J. et al. Train the trainers: Medical technology for the sustainable development of Africa. In *2018 IEEE Global Humanitarian Technology Conference (GHTC)*. 10.1109/GHTC.2018.8601677 (2018).

[60] Gandhi, M. et al. Mentoring the mentors: Implementation and evaluation of four Fogarty-sponsored mentoring training workshops in low-and middle-income countries. *Am. J. Trop. Med. Hyg.* **100**, 20–28 (2019). 30430977 10.4269/ajtmh.18-0559 PMC6329359

[61] Botero-Mesa, S. et al. Leveraging human resources for outbreak analysis: Lessons from an international collaboration to support the sub-Saharan African COVID-19 response. *BMC Public Health* **22**, 1073 (2022). 35641949 10.1186/s12889-022-13327-1 PMC9152815

