# Supplementary material for: Unleashing potential: assessing Africa’s readiness for the data science revolution to impact health
Source: Nat Commun. 2026 Apr 13;17:5138. doi: 10.1038/s41467-026-71454-4 (PMC13250160; doi:10.1038/s41467-026-71454-4)
Supplement: Supplementary file 1 — Supplementary Information [file 41467_2026_71454_MOESM1_ESM.pdf]

HPC Resources in Africa - Discovery Survey

HPC Resources in Africa - Discovery SurveyDue to the massive amounts of data produced by modern analysis methods, including high-throughput sequencing and bioinformatics, life sciences research is becoming more computational and increasingly requires access to high-performance computing (HPC) resources and HPC-compliant applications.This collaborative survey brings together the African BioGenome Project, HPC Ecosystems Project, H3ABioNet, eLwazi and DS-I Africa Consortiums as survey partners, aiming to map existing HPC infrastructures and resources on the continent for research purposes surrounding computing and analyses of various life sciences data in Africa.

Page 1 of 4

What type of HPC or compute resources do you have access to?
If you answer None or I don't know, we would still like to collect your information to identify gaps in access in Africa. (Note: High-end desktops / laptops for scientific computing do not qualify as an HPC resource.)

- ☐ HPC / data centre resources / cloud-based computing resources
- ☐ Computer lab / cluster / grid type environment for high computational workloads
- ☐ Scientific computing workstations
- ☐ Other ______
- ☐ None / I don't know

DATA PRIVACY AND CONSENT

The information you provide in this survey will be stored on UCT servers behind a firewall on secure servers and shared with restricted access only to the survey partners for ongoing communications, tracking, evaluation, research and reporting purposes specific to understanding HPC resources in Africa.

- Your personal details will be de-identified (by removal of names, email addresses and institution) whenever used in any type of reporting and will be shared securely with only the survey partners for ongoing communication if agreed to by yourself (you can indicate your preference in this regard in a question at the end of this survey).
- Your personal details will not be shared with any 3rd parties without first obtaining your explicit permission.
- Information about your institution's/organisation's HPC resources will be used for research purposes and may be published unless you indicate otherwise below. You may at any time request that your information provided be edited or removed from all of our databases (however it may not be possible to remove any data that has already been published on). To edit/remove your information or for any queries, please contact info.elwazi@uct.ac.za.

- ○ I agree
- ○ I agree, but I do not want HPC resources information I give to be publicly linked to our institution/organisation
- ○ I disagree

YOUR PERSONAL DETAILS

Title:	○ Prof<br>○ Dr<br>○ Mr<br>○ Mrs<br>○ Ms<br>○ Mx
First Name/s:	______________________
Surname:	______________________
Preferred email address for communications:	______________________

Please verify your preferred email address for communications: __________________________________

Your verification email address doesn't match

Your organisational / institutional email address if different from the above preferred email: __________________________________

City/Region of residence: __________________________________
(e.g Nairobi)

Country of residence:

- ◯ Afghanistan
- ◯ Albania
- ◯ Algeria
- ◯ American Samoa
- ◯ Andorra
- ◯ Angola
- ◯ Anguilla
- ◯ Antarctica
- ◯ Antigua and Barbuda
- ◯ Argentina
- ◯ Armenia
- ◯ Aruba
- ◯ Australia
- ◯ Austria
- ◯ Azerbaijan
- ◯ Bahamas
- ◯ Bahrain
- ◯ Bangladesh
- ◯ Barbados
- ◯ Belarus
- ◯ Belgium
- ◯ Belize
- ◯ Benin
- ◯ Bermuda
- ◯ Bhutan
- ◯ Bolivia
- ◯ Bosnia and Herzegovina
- ◯ Botswana
- ◯ Bouvet Island
- ◯ Brazil
- ◯ British Indian Ocean Terr
- ◯ Brunei
- ◯ Bulgaria
- ◯ Burkina Faso
- ◯ Burundi
- ◯ Cambodia
- ◯ Cameroon
- ◯ Canada
- ◯ Cape Verde
- ◯ Cayman Islands
- ◯ Central African Republic
- ◯ Chad
- ◯ Chile
- ◯ China
- ◯ Christmas Island
- ◯ Cocos (Keeling) Islands
- ◯ Colombia
- ◯ Comoros
- ◯ Congo
- ◯ Congo, The Democratic Rep
- ◯ Cook Islands
- ◯ Costa Rica
- ◯ Cote d'Ivoire
- ◯ Croatia
- ◯ Cuba
- ◯ Cyprus
- ◯ Czech Republic
- ◯ Denmark
- ◯ Djibouti
- ◯ Dominica
- ◯ Dominican Republic
- ◯ East Timor
- ◯ Ecuador
- ◯ Egypt
- ◯ El Salvador
- ◯ Equatorial Guinea
- ◯ Eritrea
- ◯ Estonia
- ◯ Ethiopia

◯ Falkland Islands
◯ Faroe Islands
◯ Fiji Islands
◯ Finland
◯ France
◯ French Guiana
◯ French Polynesia
◯ French Southern territori
◯ Gabon
◯ Gambia
◯ Georgia
◯ Germany
◯ Ghana
◯ Gibraltar
◯ Greece
◯ Greenland
◯ Grenada
◯ Guadeloupe
◯ Guam
◯ Guatemala
◯ Guinea
◯ Guinea-Bissau
◯ Guyana
◯ Haiti
◯ Heard Island and McDonald
◯ Holy See (Vatican City St
◯ Honduras
◯ Hong Kong
◯ Hungary
◯ Iceland
◯ India
◯ Indonesia
◯ Iran
◯ Iraq
◯ Ireland
◯ Israel
◯ Italy
◯ Jamaica
◯ Japan
◯ Jordan
◯ Kazakstan
◯ Kenya
◯ Kiribati
◯ Kuwait
◯ Kyrgyzstan
◯ Laos
◯ Latvia
◯ Lebanon
◯ Lesotho
◯ Liberia
◯ Libyan Arab Jamahiriya
◯ Liechtenstein
◯ Lithuania
◯ Luxembourg
◯ Macao
◯ Macedonia
◯ Madagascar
◯ Malawi
◯ Malaysia
◯ Maldives
◯ Mali
◯ Malta
◯ Marshall Islands
◯ Martinique
◯ Mauritania
◯ Mauritius
◯ Mayotte
◯ Mexico
◯ Micronesia, Federated Sta
◯ Moldova
◯ Monaco

◯ Mongolia
◯ Montserrat
◯ Morocco
◯ Mozambique
◯ Myanmar
◯ Namibia
◯ Nauru
◯ Nepal
◯ Netherlands
◯ Netherlands Antilles
◯ New Caledonia
◯ New Zealand
◯ Nicaragua
◯ Niger
◯ Nigeria
◯ Niue
◯ Norfolk Island
◯ North Korea
◯ Northern Mariana Islands
◯ Norway
◯ Oman
◯ Pakistan
◯ Palau
◯ Palestine
◯ Panama
◯ Papua New Guinea
◯ Paraguay
◯ Peru
◯ Philippines
◯ Pitcairn
◯ Poland
◯ Portugal
◯ Puerto Rico
◯ Qatar
◯ Reunion
◯ Romania
◯ Russian Federation
◯ Rwanda
◯ Saint Helena
◯ Saint Kitts and Nevis
◯ Saint Lucia
◯ Saint Pierre and Miquelon
◯ Saint Vincent and the Gre
◯ Samoa
◯ San Marino
◯ Sao Tome and Principe
◯ Saudi Arabia
◯ Senegal
◯ Seychelles
◯ Sierra Leone
◯ Singapore
◯ Slovakia
◯ Slovenia
◯ Solomon Islands
◯ Somalia
◯ South Africa
◯ South Georgia and the Sou
◯ South Korea
◯ South Sudan
◯ Spain
◯ Sri Lanka
◯ Sudan
◯ Suriname
◯ Svalbard and Jan Mayen
◯ Swaziland
◯ Sweden
◯ Switzerland
◯ Syria
◯ Taiwan
◯ Tajikistan
◯ Tanzania

◯ Thailand
◯ Togo
◯ Tokelau
◯ Tonga
◯ Trinidad and Tobago
◯ Tunisia
◯ Turkey
◯ Turkmenistan
◯ Turks and Caicos Islands
◯ Tuvalu
◯ Uganda
◯ Ukraine
◯ United Arab Emirates
◯ United Kingdom
◯ United States
◯ United States Minor Outly
◯ Uruguay
◯ Uzbekistan
◯ Vanuatu
◯ Venezuela
◯ Vietnam
◯ Virgin Islands, British
◯ Virgin Islands, U.S.
◯ Wallis and Futuna
◯ Western Sahara
◯ Yemen
◯ Yugoslavia
◯ Zambia
◯ Zimbabwe

YOUR ORGANISATION /INSTITUTION DETAILS

Please provide your institution's / organisation's name:	______________________________<br>(e.g. University of Cape Town; African BioGenome Project)
Type of Institution:	◯ Academia/Research organization<br>◯ Industry/Private sector<br>◯ National or government facility<br>◯ Other ______
Position / Role in your institution/organisation:	◯ Leadership / management<br>◯ Academic / Researcher<br>◯ Technical<br>◯ Student<br>◯ Other ______
Please provide your job title:	______________________________
URL to your institution/organisation department:	______________________________
City/Region where your institution/organisation is located:	______________________________<br>(e.g Nairobi)

Country base of institution/organisation:

◯ Afghanistan
◯ Albania
◯ Algeria
◯ American Samoa
◯ Andorra
◯ Angola
◯ Anguilla
◯ Antarctica
◯ Antigua and Barbuda
◯ Argentina
◯ Armenia
◯ Aruba
◯ Australia
◯ Austria
◯ Azerbaijan
◯ Bahamas
◯ Bahrain
◯ Bangladesh
◯ Barbados
◯ Belarus
◯ Belgium
◯ Belize
◯ Benin
◯ Bermuda
◯ Bhutan
◯ Bolivia
◯ Bosnia and Herzegovina
◯ Botswana
◯ Bouvet Island
◯ Brazil
◯ British Indian Ocean Terr
◯ Brunei
◯ Bulgaria
◯ Burkina Faso
◯ Burundi
◯ Cambodia
◯ Cameroon
◯ Canada
◯ Cape Verde
◯ Cayman Islands
◯ Central African Republic
◯ Chad
◯ Chile
◯ China
◯ Christmas Island
◯ Cocos (Keeling) Islands
◯ Colombia
◯ Comoros
◯ Congo
◯ Congo, The Democratic Rep
◯ Cook Islands
◯ Costa Rica
◯ Cote d'Ivoire
◯ Croatia
◯ Cuba
◯ Cyprus
◯ Czech Republic
◯ Denmark
◯ Djibouti
◯ Dominica
◯ Dominican Republic
◯ East Timor
◯ Ecuador
◯ Egypt
◯ El Salvador
◯ Equatorial Guinea
◯ Eritrea
◯ Estonia
◯ Ethiopia

◯ Falkland Islands
◯ Faroe Islands
◯ Fiji Islands
◯ Finland
◯ France
◯ French Guiana
◯ French Polynesia
◯ French Southern territori
◯ Gabon
◯ Gambia
◯ Georgia
◯ Germany
◯ Ghana
◯ Gibraltar
◯ Greece
◯ Greenland
◯ Grenada
◯ Guadeloupe
◯ Guam
◯ Guatemala
◯ Guinea
◯ Guinea-Bissau
◯ Guyana
◯ Haiti
◯ Heard Island and McDonald
◯ Holy See (Vatican City St
◯ Honduras
◯ Hong Kong
◯ Hungary
◯ Iceland
◯ India
◯ Indonesia
◯ Iran
◯ Iraq
◯ Ireland
◯ Israel
◯ Italy
◯ Jamaica
◯ Japan
◯ Jordan
◯ Kazakstan
◯ Kenya
◯ Kiribati
◯ Kuwait
◯ Kyrgyzstan
◯ Laos
◯ Latvia
◯ Lebanon
◯ Lesotho
◯ Liberia
◯ Libyan Arab Jamahiriya
◯ Liechtenstein
◯ Lithuania
◯ Luxembourg
◯ Macao
◯ Macedonia
◯ Madagascar
◯ Malawi
◯ Malaysia
◯ Maldives
◯ Mali
◯ Malta
◯ Marshall Islands
◯ Martinique
◯ Mauritania
◯ Mauritius
◯ Mayotte
◯ Mexico
◯ Micronesia, Federated Sta
◯ Moldova
◯ Monaco

◯ Mongolia
◯ Montserrat
◯ Morocco
◯ Mozambique
◯ Myanmar
◯ Namibia
◯ Nauru
◯ Nepal
◯ Netherlands
◯ Netherlands Antilles
◯ New Caledonia
◯ New Zealand
◯ Nicaragua
◯ Niger
◯ Nigeria
◯ Niue
◯ Norfolk Island
◯ North Korea
◯ Northern Mariana Islands
◯ Norway
◯ Oman
◯ Pakistan
◯ Palau
◯ Palestine
◯ Panama
◯ Papua New Guinea
◯ Paraguay
◯ Peru
◯ Philippines
◯ Pitcairn
◯ Poland
◯ Portugal
◯ Puerto Rico
◯ Qatar
◯ Reunion
◯ Romania
◯ Russian Federation
◯ Rwanda
◯ Saint Helena
◯ Saint Kitts and Nevis
◯ Saint Lucia
◯ Saint Pierre and Miquelon
◯ Saint Vincent and the Gre
◯ Samoa
◯ San Marino
◯ Sao Tome and Principe
◯ Saudi Arabia
◯ Senegal
◯ Seychelles
◯ Sierra Leone
◯ Singapore
◯ Slovakia
◯ Slovenia
◯ Solomon Islands
◯ Somalia
◯ South Africa
◯ South Georgia and the Sou
◯ South Korea
◯ South Sudan
◯ Spain
◯ Sri Lanka
◯ Sudan
◯ Suriname
◯ Svalbard and Jan Mayen
◯ Swaziland
◯ Sweden
◯ Switzerland
◯ Syria
◯ Taiwan
◯ Tajikistan
◯ Tanzania

◯ Thailand
◯ Togo
◯ Tokelau
◯ Tonga
◯ Trinidad and Tobago
◯ Tunisia
◯ Turkey
◯ Turkmenistan
◯ Turks and Caicos Islands
◯ Tuvalu
◯ Uganda
◯ Ukraine
◯ United Arab Emirates
◯ United Kingdom
◯ United States
◯ United States Minor Outly
◯ Uruguay
◯ Uzbekistan
◯ Vanuatu
◯ Venezuela
◯ Vietnam
◯ Virgin Islands, British
◯ Virgin Islands, U.S.
◯ Wallis and Futuna
◯ Western Sahara
◯ Yemen
◯ Yugoslavia
◯ Zambia
◯ Zimbabwe

Please indicate which communities you have an affiliation with:

☐ African BioGenome Project
☐ DS-I Africa
☐ eLwazi
☐ H3ABioNet
☐ HPC Ecosystems Project
☐ Other ______
☐ Not applicable

HPC Resources in Africa - Technical Information

HPC RESOURCE Page 2 of 4

Please fill in this page with as much information as possible for a single HPC Resource that you have access to (i.e. you have a login to a local or remote system).

You will have an opportunity at the end of the page to submit an additional HPC Resource if you have access to more than one.

If you are unable to complete the Technical Details section for this HPC Resource, that's okay as not all HPC users will know this level of detail and the fields are optional.

Name of HPC (if known): ______________________________

URL to the HPC information page: ______________________________
(E.g. a user guide; wiki page; landing page etc.)

Where is the HPC / compute resource hosted?

- ◯ On premise (your institution's local site)
- ◯ Remote premise (your institution but not a local site)
- ◯ Remote premise (another institution's site)
- ◯ Cloud platform
- ◯ Other ______
- ◯ I don't know

Cloud based platform being used:

- ◯ AWS
- ◯ GCP
- ◯ Azure
- ◯ Other {cloud_platform_other}

Is your HPC / compute resource available for collaboration?

- ◯ Yes
- ◯ No
- ◯ I don't know

If yes, please check the different levels of collaboration:

- ☐ Departmental resource/use
- ☐ Organizational use
- ☐ Project based collaborations
- ☐ National use
- ☐ Geographical or continental use
- ☐ Available for external usage based on prearranged agreement/authority
- ☐ I don't know

Do you currently have capacity to host additional scientific computing resources?
(i.e. infrastructure, personnel, support equipment, etc.)

- ◯ Yes
- ◯ No
- ◯ No, but we would like to be contacted about this
- ◯ I don't know

What are your current computational, data transfer challenges? ______________________________

HPC RESOURCE: Technical Details

Are you the contact person or systems administrator for this HPC resource?	◯ Yes<br>◯ No<br>◯ No, but I can provide his/her publicly available contact details
Year of HPC Resource deployment:	◯ 2024<br>◯ 2023<br>◯ 2022<br>◯ 2021<br>◯ 2020<br>◯ 2019<br>◯ 2018<br>◯ 2017<br>◯ 2016<br>◯ Prior to 2016<br>◯ Unknown
Do you intend on replacing your HPC within the next 12 months?	◯ Yes<br>◯ No<br>◯ I don't know

HPC Resource Technical Details:

Please complete all details you have knowledge of with regards to this HPC Resource.

Total number of compute nodes: ______
Number of cores per node: ______
Total number of shared cores across HPC resource: ______
Memory per core (GB): ______
Memory per node (GB): ______
Total shared memory across HPC resource (GB): ______
Does the system have GPU/s? ______
Any alternative accelerators? ______

HPC RESOURCE: System Administrator or Contact Person

Name and surname of the contact person at that institution/organisation:	________________________________
Email address of the contact person at that institution/organisation:	________________________________
If possible, provide telephone number of the contact person at that institution/organisation:	________________________________<br>(Please provide country code e.g. +27211231234)

HPC Resources in Africa - Skills

HPC INFORMATICS & TECHNICAL SKILLS Page 3 of 4

What informatics skill sets are available within your organization/division?	☐ Bioinformatics<br>☐ Taxonomy<br>☐ Proteomics<br>☐ Data Science<br>☐ Machine Learning<br>☐ Genome assembly<br>☐ Genome annotation<br>☐ Genome analysis<br>☐ Other ______<br>☐ I don't know
What type of technical support skills do you have within your organization?	☐ Linux System Administration<br>☐ Windows System Administration<br>☐ Networking<br>☐ Storage<br>☐ HPC support skills<br>☐ Other ______<br>☐ I don't know
Please indicate how these informatics / technical support skills are available for collaboration:	☐ Departmental resource/use<br>☐ Organizational use<br>☐ Project based collaboration<br>☐ Available for external usage based on prearranged agreement/authority<br>☐ I don't know
What domain of informatics does your organisation / institution conduct?	☐ Plant<br>☐ Animal<br>☐ Microbial<br>☐ Human<br>☐ Other ______

HPC Resources in Africa - Final

OTHER HPC Page 4 of 4

Are you aware of the "South African CHPC's HPC Ecosystems Project"?

◯ Yes
◯ No

Would you like to join the HPC Ecosystems Project slack community?

The HPC Ecosystems Project performs HPC system deployments and HPC system administrator training to partner institutions in Africa and is committed to building a community of HPC practitioners in Africa. ecosystems.nicis.ac.za

◯ Yes
◯ No
◯ I have already joined

Do you know about or have you used HPC facilities at any other institution / organization in Africa?

◯ Yes
◯ No

Please provide publicly available contact details for the other HPC Resources you know of:

(if you do not have publicly available contact details you can proceed to the next section)

Institution / Organisation	Contact's Name	Email address	Contact Number
______	______	______	______
______	______	______	______
______	______	______	______
______	______	______	______

FINAL COMMENTS

Can we follow up with you based on your feedback in this survey?

◯ Yes
◯ No

Any other comments or queries?

____
