# Supplementary material for: Unleashing potential: assessing Africa’s readiness for the data science revolution to impact health
Source: Nat Commun. 2026 Apr 13;17:5138. doi: 10.1038/s41467-026-71454-4 (PMC13250160; doi:10.1038/s41467-026-71454-4)
Supplement: Supplementary file 2 — Description of Additional Supplementary Files [file 41467_2026_71454_MOESM2_ESM.pdf]

Description of Additional Supplementary Files

File Name: Supplementary Data 1
Description: List of example Cloud provider certifications

File Name: Supplementary Data 2
Description: Data Science related degree programs at African institutions.
