# Supplementary material for: Unleashing potential: assessing Africa’s readiness for the data science revolution to impact health
Source: Nat Commun. 2026 Apr 13;17:5138. doi: 10.1038/s41467-026-71454-4 (PMC13250160; doi:10.1038/s41467-026-71454-4)
Supplement: Supplementary file 4 — Supplementary Data 2 [file 41467_2026_71454_MOESM4_ESM.pdf]

Supplementary Data 2. Data Science related degree programs at African institutions.

Region	School	Country	Degree programmes/Academic Qualification	Faculty	Direct URL	Institution URL	Date URL Accessed
Eastern Africa	Addis Ababa University	Ethiopia	MSc. Information Science And Systems (Health Information/ Information Science/Information System)			Http://Www.Aau.Edu.Et/Cns/School-Of-Information-Science/	05/06/2024
Eastern Africa	African Institute For Mathematical Sciences (Aims) In Collaboration With Quantum Leap Africa (Qla)	Rwanda	PhD In Data Science		Https://Quantumleapafrica.Org/Course/PhD-In-Data-Science-2023/		04/06/2024
Eastern Africa	Jamhuriya University Of Science And Technology	Somalia	MSc Data Science			Https://Just.Edu.So/Course/Master-Of-Science-In-Data-Science/	05/06/2024
Eastern Africa	Jomo Kenyatta University Of Agriculture And Technology	Kenya	MSc. Mathematics (Data Science)			Https://Www.Jkuat.Ac.Ke/Jkuat-Launches-New-Course-In-Data-Science-And-Analytics/	05/06/2024
Eastern Africa	Jomo Kenyatta University Of Agriculture And Technology	Kenya	BSc Computer Science		Https://Www.Jkuat.Ac.Ke/Undergraduate-Courses/BSc-Computer-Science/		04/06/2024
Eastern Africa	Jomo Kenyatta University Of Agriculture And Technology	Kenya	BSc Information Technology		Https://Www.Jkuat.Ac.Ke/Undergraduate-Courses/BSc-Information-Technology/		04/06/2024
Eastern Africa	Makerere University	Uganda	AI & Data Science		Https://Cs.Mak.Ac.Ug/Research/Groups_And_Centers		05/06/2024

Eastern Africa	Makerere University	Uganda	MSc. In Computer Science	Computer Science	Https://Cs.Mak.Ac.Ug/Admission/Prospective-Graduate-Students		10/06/2024
Eastern Africa	Makerere University	Uganda	PhD In Computer Science	Computer Science	Https://Cs.Mak.Ac.Ug/Admission/Prospective-PhD-Students		10/06/2024
Eastern Africa	Moi University (University Of New York)	Kenya	Master Of Science In Information Technology		Https://Is.Mu.Ac.Ke/Index.Php/K2-Categories/Masters-Is/MSc-In-Information-Technology		10/06/2024
Eastern Africa	Moi University (University Of New York)	Kenya	(PhD) In Communication Studies		Https://Is.Mu.Ac.Ke/Index.Php/Doctorate/Dphil-In-Communication-Studies		10/06/2024
Eastern Africa	National Institute Of Statistics	Rwanda				Https://Statistics.Gov.Rw/Content/Data-Revolution/Nisr-Training-Centre	05/06/2024
Eastern Africa	Strathmore University (Kenya)	Kenya	BScIn Informatics And Computer Science (BSc. Ics)		Https://Strathmore.Edu/BSc-In-Informatics-And-Computer-Science/		04/06/2024
Eastern Africa	Strathmore University (Kenya)	Kenya	BScIn Statistics And Data Science		Https://Strathmore.Edu/BSc-Statistics-And-Data-Science/		04/06/2024
Eastern Africa	Strathmore University (Kenya)	Kenya	Master Of Science In Data Science And Analytics		Https://Strathmore.Edu/MSc-Data-Science-And-Analytics/		04/06/2024
Eastern Africa	The African Centre Of Excellence In Data Science (Ace-Ds)<br>National University Of Rwanda (Nur)	Rwanda	The Master's Degree With Specialization In Data Mining		Https://Aceds.Ur.Ac.Rw/Specialization-Data-Mining		05/06/2024
Eastern Africa	University Of Dar Es Salaam	Tanzania	MSc Data Science		Https://Cse.Udsm.Ac.Tz/Programs/Program-Details/MSc-In-Data-Science-%28MSc-Dsc%29#:~:Text=This%20is%20a%		05/06/2024

					20two%2dyear,Evolving%20data%2ddriven%20business%20world.		
Eastern Africa	University Of Nairobi	Kenya	BSc Computer Science		Https://Computerscience.Uonbi.Ac.Ke/Admission-Content-Type/Bachelor-Science-Computer-Science		04/06/2024
Eastern Africa	University Of Nairobi (Columbia University)	Kenya	Master Of Science In Data Science		Https://Computerscience.Uonbi.Ac.Ke/Index.Php/Admission-Content-Type/Master-Science-Computer-Science		10/06/2024
Eastern Africa	University Of Nairobi (Columbia University)	Kenya	Master Of Science In Information Systems		Https://Computerscience.Uonbi.Ac.Ke/Admission-Content-Type/Master-Science-Information-Systems		10/06/2024
Eastern Africa	University Of Nairobi (Columbia University)	Kenya	Doctor Of Philosophy In Information Systems		Https://Computerscience.Uonbi.Ac.Ke/Admission-Content-Type/Doctor-Philosophy-Information-Systems		10/06/2024
Eastern Africa	University Of Rwanda (Washington University St Louis)	Rwanda	Masters Of Science In Information Systems	Science And Technology		Https://Ur.Ac.Rw/?Postgraduate	10/06/2024
Eastern Africa	University Of Rwanda (Washington University St Louis)	Rwanda	PhD In Computer Science	Science And Technology		Https://Ur.Ac.Rw/?Postgraduate	10/06/2024
Southern Africa	Botswana International University Of Science And Technology	Botswana	BSc Information Systems & Data Management	Science	Https://Www.Biust.Ac.Bw/Biust-Programmes/BSc-Information-Systems-And-Data-Management/		04/06/2024
Southern Africa	Botswana International University Of Science And Technology	Botswana	BSc- Computer Science And Software Engineering	Science	Https://Www.Biust.Ac.Bw/Biust-Programmes/BSc-		04/06/2024

					Computer-Science-And-Software-Engineering/		
Southern Africa	Botswana International University Of Science And Technology	Botswana	Master Of Science Degree: Computer Science	Science	Https://Www.Biust.Ac.Bw/Post-Graduate-Programmes/		04/06/2024
Southern Africa	Botswana International University Of Science And Technology	Botswana	Master Of Science Degree: Information Systems	Science	Https://Www.Biust.Ac.Bw/Post-Graduate-Programmes/		04/06/2024
Southern Africa	Botswana International University Of Science And Technology	Botswana	Doctoral Science Degrees: Computer Science And Software Engineering	Science	Https://Www.Biust.Ac.Bw/Post-Graduate-Programmes/		04/06/2024
Southern Africa	Botswana International University Of Science And Technology	Botswana	Doctoral Science Degrees: Information Systems	Science	Https://Www.Biust.Ac.Bw/Post-Graduate-Programmes/		04/06/2024
Southern Africa	Chinhoyi University Of Zimbabwe	Zimbabwe	MSc Data Analytics			Https://Www.Cut.Ac.Zw/Welcome/Admissions	05/06/2024
Southern Africa	Eden University	Zambia	MSc Data Analytics			Https://Www.Edenuniversity.Edu.Zm/Courses/Become-A-Php-Master-And-Make-Money-Fast/	04/06/2024
Southern Africa	Namibia University Of Science And Technology	Namibia	MSc Applied Statistics	Health, Natural Resources And Applied Sciences		Https://Www.Nust.Na/Programmes/Master-Science-Applied-Statistics	05/06/2024
Southern Africa	Namibia University Of Science And Technology	Namibia	Bachelor Of Computer Science	Computing And Informatics	Https://Www.Nust.Na/Programmes/Bachelor-Computer-Science		04/06/2024
Southern Africa	Namibia University Of Science And Technology	Namibia	BSc Applied Mathematics And Statistics	Health, Natural Resources And Applied Sciences	Https://Www.Nust.Na/Programmes/Bachelor-Science-Applied-Maths-Statistics		04/06/2024

Southern Africa	North West University	South Africa	BSc, MSc, PhD Pure And Applied Analytics			Https://Natural-Sciences.Nwu.Ac.Za/Paa	05/06/2024
Southern Africa	North West University	South Africa	BSc Data Mining/Data Science		Https://Natural-Sciences.Nwu.Ac.Za/Data-Mining	https://natural-sciences.nwu.ac.za/centre-business-mathematics-and-informatics/qualifications	05/06/2024
Southern Africa	Rhodes University	South Africa	MSc Bioinformatics			Https://Rubi.Ru.Ac.Za/	05/06/2024
Southern Africa	Sol Plaatje University	South Africa	BSc Data Science	Natural & Applied Sciences	Https://Www.Spu.Ac.Za/Index.Php/Spu-Nas-Programmes/		04/06/2024
Southern Africa	Sol Plaatje University	South Africa	BSc (Hons) Computer Science	Natural & Applied Sciences	Https://Www.Spu.Ac.Za/Index.Php/Spu-Nas-Programmes/		04/06/2024
Southern Africa	Sol Plaatje University	South Africa	BSc (Hons) Data Science	Natural & Applied Sciences	Https://Www.Spu.Ac.Za/Index.Php/Spu-Nas-Programmes/		04/06/2024
Southern Africa	Unisa (University Of South Africa)	South Africa	BSc Chemistry And Computer Science		Https://Www.Unisa.Ac.Za/Sites/Corporate/Default/Register-To-Study-Through-Unisa/Undergraduate-&-Honours-Qualifications/Find-Your-Qualification-&-Choose-Your-Modules/All-Qualifications/Bachelor-Of-Science-Chemistry-And-Computer-Science-(98801-%E2%80%93-Ccs)		04/06/2024
Southern Africa	Unisa (University Of South Africa)	South Africa	BSc Mathematics And Computer Science		Https://Www.Unisa.Ac.Za/Sites/Corporate/Default/Register-To-Study-		04/06/2024

					Through-Unisa/Undergraduate-&-Honours-Qualifications/Find-Your-Qualification-&-Choose-Your-Modules/All-Qualifications/Bachelor-Of-Science-Applied-Mathematics-And-Computer-Science-(98801-%E2%80%93-Amc)		
Southern Africa	Unisa (University Of South Africa)	South Africa	BSc Computing		Https://Www.Unisa.Ac.Za/Sites/Corporate/Default/Register-To-Study-Through-Unisa/Undergraduate-&-Honours-Qualifications/Find-Your-Qualification-&-Choose-Your-Modules/All-Qualifications/Bachelor-Of-Science-In-Computing-(98906-%E2%80%93-Com)		04/06/2024
Southern Africa	Unisa (University Of South Africa)	South Africa	BSc Informatics		Https://Www.Unisa.Ac.Za/Sites/Corporate/Default/Register-To-Study-Through-Unisa/Undergraduate-&-Honours-Qualifications/Find-Your-Qualification-&-Choose-Your-Modules/All-Qualifications/Bachelor-Of-Science-In-		04/06/2024

					Informatics-(98907-%E2%80%93-Inf)		
Southern Africa	Unisa (University Of South Africa)	South Africa	BSc Honours In Computing		Https://Www.Unisa.Ac.Za/Sites/Corporate/Default/Register-To-Study-Through-Unisa/Undergraduate-&-Honours-Qualifications/Find-Your-Qualification-&-Choose-Your-Modules/All-Qualifications/Bachelor-Of-Science-Honours-In-Computing-(98908)		04/06/2024
Southern Africa	Unisa (University Of South Africa)	South Africa	Master Of Science In Computing	Science	Https://Www.Unisa.Ac.Za/Sites/Corporate/Default/Apply-For-Admission/Master%27s-&-Doctoral-Degrees/Qualifications/All-Qualifications/Master-Of-Science-In-Computing-(98961)		04/06/2024
Southern Africa	Unisa (University Of South Africa)	South Africa	Doctor Of Philosophy In Computer Science		Https://Www.Unisa.Ac.Za/Sites/Corporate/Default/Apply-For-Admission/Master%27s-&-Doctoral-Degrees/Qualifications/All-Qualifications/Doctor-Of-Philosophy-In-Computer-Science-(98803)		04/06/2024
Southern Africa	Unisa (University Of South Africa)	South Africa	Doctor Of Philosophy In Information Systems		Https://Www.Unisa.Ac.Za/Sites/Corporate/Default/Apply-For-		04/06/2024

					Admission/Master%27s-&-Doctoral-Degrees/Qualifications/All-Qualifications/Doctor-Of-Philosophy-In-Information-Systems-(98804)		
Southern Africa	University Of Cape Town	South Africa	Masters Programmes In Data Science			Https://Www.Gsb.Uct.Ac.Za/Data-Science-Leadership/?Utc_Source=Google&Utc_Medium=Cpc&Autc_Campaign=20276618877&Utc_Term=Data%20science%20courses&Gclid=Cjwkcajw44mlbhaqeiwaQp3evparpl_Ilyfkvzexaw7d2wwygt7ollut90aihgfv6rat_4nq_Yr4jbocnloqavd_Bwe	05/06/2024
Southern Africa	University Of Cape Town	South Africa	BScIn Computer Science With A Specialization In Data Science	Science	Https://Science.Uct.Ac.Za/Departments/School-It-Incorporating-Computer-Science-And-Information-Systems		04/06/2024
Southern Africa	University Of Cape Town	South Africa	MSc Data Science		Https://Science.Uct.Ac.Za/Departments/School-It-Incorporating-Computer-Science-And-Information-Systems		04/06/2024
Southern Africa	University Of Cape Town	South Africa	PhD In Computer Science With A Specialization In Data Science		Https://Science.Uct.Ac.Za/Departments/School-It-Incorporating-		04/06/2024

					Computer-Science-And-Information-Systems		
Southern Africa	University Of Johannesburg	South Africa	Academy Of Computer Science And Software Engineering	Science	Https://Www.Uj.Ac.Za/Faculties/Science/Courses-And-Programmes/Short-Courses/		04/06/2024
Southern Africa	University Of Kwazulu- Natal	South Africa	BSc Data Science (Combination Of Computer Science & Statistics)	Mathematics, Statistics & Computer Science		Https://SMScs.Ukzn.Ac.Za/Data-Science/	05/06/2024
Southern Africa	University Of Kwazulu- Natal	South Africa	BSc Computer Science And Information Technology	Mathematics, Statistics And Computer Science		Https://SMScs.Ukzn.Ac.Za/Computer-Science/	04/06/2024
Southern Africa	University Of Kwazulu- Natal	South Africa	Masters Degree In Data Science	Mathematics, Statistics And Computer Science	Https://SMScs.Ukzn.Ac.Za/Wp-Content/Uploads/2021/11/2021-Data-Science-Degree-Poster.Pdf		11/06/24
Southern Africa	University Of Kwazulu- Natal (T.H Chan Sch Of Public Health, Heidelberg Institute Of Global Health)	South Africa	Master In Medical Science (HIV Pathogenesis Programme)	Health Sciences	Https://Ww3.Chs.Ukzn.Ac.Za/Postgraduate-Program/Master-In-Medical-Science-Hiv-Pathogenesis-Programme/		11/06/24
Southern Africa	University Of Pretoria	South Africa	BSc Computer Science		Https://Www.Up.Ac.Za/Computer-Science/Article/1972602/Undergraduate-Degrees		04/06/2024
Southern Africa	University Of Pretoria	South Africa	MIT Big Data Science		Https://Www.Up.Ac.Za/School-Of-Information-Technology/Article/2324622/Mit-In-Big-Data-Science-Stream-C		04/06/2024

Southern Africa	Stellenbosch University	South Africa	Bachelor Of Data Science (Bdatsci)		Https://Www.Sun.Ac.Za/English/Data-Science-And-Computational-Thinking/Academics/Careers-In-Data-Science-And-Computational-Thinking		04/06/2024
Southern Africa	Stellenbosch University	South Africa	BSc In Computer Science	Science	Https://Www.Sun.Ac.Za/English/Faculty/Science/Pages/Undergraduate-Mathematical.Aspx#Cs		04/06/2024
Southern Africa	Stellenbosch University	South Africa	PhD In Computer Science With A Specialization In Data Science		Https://Www.Wits.Ac.Za/Course-Finder/Postgraduate/Science/MSc-Data-Science/		04/06/2024
Southern Africa	Stellenbosch University*	South Africa	MSc Computer Science (Data Science)*			Https://Www.Cs.Sun.Ac.Za/Teaching/Masters/#:~:Text=Our%20masters%20degree%20is%20a,Edge	05/06/2024
Southern Africa	University Of The Western Cape	South Africa	BSc (Bachelor Of Science) In Mathematics			Https://Www.Uwc.Ac.Za/Study/Faculties-And-Programmes/Faculty-Of-Natural-Sciences/Programmes	04/06/2024
Southern Africa	University Of The Western Cape	South Africa	BSc (Bachelor Of Science) In Computer Science			Https://Www.Uwc.Ac.Za/Study/Faculties-And-Programmes/Faculty-Of-Natural-Sciences/Programmes	04/06/2024
Southern Africa	University Of The Western Cape	South Africa	MSc (Master's) In Statistics			Https://Www.Uwc.Ac.Za/Study/Facultie	04/06/2024

						s-And-Programmes/Faculty-Of-Natural-Sciences/Programmes	
Southern Africa	University Of The Witwatersrand	South Africa	BSc Computer Science	Science	Https://Www.Wits.Ac.Za/Course-Finder/Undergraduate/Science/Computer-Science/		04/06/2024
Southern Africa	University Of The Witwatersrand	South Africa	MSc Computer Science - Coursework	Science	Https://Www.Wits.Ac.Za/Course-Finder/Postgraduate/Science/MSc-Computer-Science/		04/06/2024
Southern Africa	University Of The Witwatersrand	South Africa	MSc Robotics	Science	Https://Www.Wits.Ac.Za/Course-Finder/Postgraduate/Science/MSc-Robotics/		04/06/2024
Southern Africa	University Of The Witwatersrand	South Africa	PhD Computational And Applied Mathematics	Science	Https://Www.Wits.Ac.Za/Course-Finder/Postgraduate/Science/PhD-Computational-And-Applied-Mathematics/		04/06/2024
Southern Africa	University Of The Witwatersrand *	South Africa	MSc Data Science *	Science		Https://Www.Wits.Ac.Za/MSc-Data-Science/?Gclid=Cjwkcajw44mlbhaqeiwaqp3evvdnd38bfnlx6kda-	05/06/2024
Southern Africa	University Of The Witwatersrand *	South Africa	BSc Big Data Analytics *	Science		Https://Www.Wits.Ac.Za/Course-Finder/Postgraduate/Science/Big-Data-Analytics/	05/06/2024
Southern Africa	University Of The Witwatersrand*	South Africa	MA E-Science *	Science			05/06/2024

Southern Africa	University Of The Witwatersrand *	South Africa	MSc Artificial Intelligence *	Science		?Gclid=Cjwkcajw44mlbhaqeiwaqp3evqgae6a6zctuhsbzkoyplpb5otebyhkjubg-Bqhhny4ssvxnpy2pzxocr6yqavd_Bwe	05/06/2024
Southern Africa	University Of The Witwatersrand *	South Africa	MSc E-Science *	Science		Https://Www.Wits.Ac.Za/Course-Finder/Postgraduate/Science/E-Science/?Gclid=Cjwkcajw44mlbhaqeiwaqp3evqtp26nnk6q_Knuwgx68j8h41ru7plcccabx4kotuehngsyog1zpfhocs4iqavd_Bwe	05/06/2024
Southern Africa	University Of Zimbabwe	Zimbabwe	BSc Artificial Intelligence And Machine Learning	Computer Engineering Informatics And Communications	Http://Www.Emhare.Uz.Ac.Zw/	Https://Www.Uz.Ac.Zw/Index.Php/Computer-Eng-Prog	05/06/2024
Southern Africa	University Of Zimbabwe	Zimbabwe	BSc Data Science And Informatics	Computer Engineering Informatics And Communications	Http://Www.Emhare.Uz.Ac.Zw/	Https://Www.Uz.Ac.Zw/Index.Php/Computer-Eng-Prog	05/06/2024
Southern Africa	University Of Zimbabwe	Zimbabwe	BSc Data Science And Systems	Computer Engineering Informatics And Communications	Http://Www.Emhare.Uz.Ac.Zw/	Https://Www.Uz.Ac.Zw/Index.Php/Computer-Eng-Prog	05/06/2024
Southern Africa	University Of Zimbabwe	Zimbabwe	MSc Bioinformatics And Genomics	Computer Engineering Informatics And	Http://Www.Emhare.Uz.Ac.Zw/	Https://Www.Uz.Ac.Zw/Index.Php/Computer-Eng-Prog	05/06/2024

				Communications			
Southern Africa	University Of Zimbabwe	Zimbabwe	MSc Data Science And Informatics	Computer Engineering Informatics And Communications	Http://Www.Emhare.Uz.Ac.Zw/	Https://Www.Uz.Ac.Zw/Index.Php/Computer-Eng-Prog	05/06/2024
Southern Africa	University Of Zimbabwe	Zimbabwe	MSc Genomics And Biotechnology	Computer Engineering Informatics And Communications	Http://Www.Emhare.Uz.Ac.Zw/	Https://Www.Uz.Ac.Zw/Index.Php/Computer-Eng-Prog	05/06/2024
Western Africa	African Institute Of Mathematical Sciences	Ghana	Big Data Analytics (short term training)			Https://Nexteinstein.Org/Industry-Initiative-2/Bd4d-Scp1-Aims-Cameroon/	05/06/2024
Western Africa	African University Of Science & Technology	Nigeria	MSc Geoinformatics & Gis		Https://Aust.Edu.Ng/Programs/School-Of-Science-And-Science-Education-1/Geoinformatics-And-Gis	Https://Aust.Edu.Ng/Programs/Computer-Science	05/06/2024
Western Africa	American University Of Nigeria	Nigeria	MSc Data Science And Analytics	Information Technology & Computing	Https://Www.Aun.Edu.Ng/Index.Php/Sitc/MSc-Data-Science		05/06/2024
Western Africa	Babcock University	Nigeria	MSc Computer Science	Science	Https://Www.Babcock.Edu.Ng/Postgraduate/Program/MSc-Computer-Science		04/06/2024
Western Africa	Lead City University	Nigeria	MSc Computer Science (Computational Intelligence)			Https://Lcu.Edu.Ng/Index.Php/2-Uncategorised/165-Csc-Courseware	05/06/2024

Western Africa	University Of Cape Coast	Ghana	MSc Data Management And Analysis			Https://Cdamaa.Ucc.Edu.Gh/Training/MSc_Data_Analysis.Html	05/06/2024
Western Africa	University Of Ibadan	Nigeria	PhD Computer Science (Data Mining)	Computer Science		Http://Sci.Ui.Edu.Ng/Compsciwelcome	05/06/2024
Western Africa	University Of Uyo	Nigeria	MPhil/PhD Computational Intelligence	Computer Science	Https://Www.Uniuyo.Edu.Ng/Index.Html	Https://Uniuyo.Edu.Ng/Faculty-Of-Science/Computer-Science/	05/06/2024
Western Africa	Kwame Nkrumah University Of Science & Technology (University Of Washington)	Ghana	BSc Computer Science (Parallel)	Computer Science	Https://Cs.Knust.Edu.Gh/Undergraduate/BSc-Computer-Science-Parallel		10/06/2024
Western Africa	Kwame Nkrumah University Of Science & Technology (University Of Washington)	Ghana	BSc Computer Science	Computer Science	Https://Cs.Knust.Edu.Gh/Undergraduate/BSc-Computer-Science		10/06/2024
Western Africa	Kwame Nkrumah University Of Science & Technology (University Of Washington)	Ghana	MSc/MPhil Information Technology	Computer Science	Https://Cs.Knust.Edu.Gh/Graduate/MScmphil-Information-Technology		10/06/2024
Western Africa	Kwame Nkrumah University Of Science & Technology (University Of Washington)	Ghana	MSc/MPhil Computer Science	Computer Science	Https://Cs.Knust.Edu.Gh/Graduate/MScmphil-Computer-Science		10/06/2024
Western Africa	Kwame Nkrumah University Of Science & Technology (University Of Washington)	Ghana	PhD Computer Science	Computer Science		Https://Cs.Knust.Edu.Gh/Postgraduate	10/06/2024
Western Africa	Kwame Nkrumah University Of Science & Technology (University Of Washington)	Ghana	PhD Information Technology	Computer Science		Https://Cs.Knust.Edu.Gh/Postgraduate	10/06/2024
Northern Africa	Ain Shams University	Egypt	BSc Physics / Computer Science	Computer And	Https://Www.Asu.Edu.Eg/436/Page#:~:Text=Ph		04/06/2024

				Information Sciences	ysics%20/%20computer %20science		
Northern Africa	Ain Shams University	Egypt	MSc Computer Science	Science	Https://Www.Asu.Edu.Eg/730/Page		04/06/2024
Northern Africa	Ain Shams University	Egypt	Doctor Of Philosophy Of Science In Computer Science	Science	Https://Www.Asu.Edu.Eg/343/Page/Doctorate-Programs		04/06/2024
Northern Africa	Al Akhawayn University (Morocco)	Morocco	BSc Computer Systems	Science	Https://Aui.Ma/Bachelor-Of-Science-In-Computer-Systems		04/06/2024
Northern Africa	Al Akhawayn University (Morocco)	Morocco	BSc Computer Science	Science	Https://Aui.Ma/Bachelor-Of-Science-In-Computer-Science		04/06/2024
Northern Africa	Al Akhawayn University (Morocco)	Morocco	MSc Big Data Analytics	Science	Https://Aui.Ma/Master-Of-Science-In-Big-Data-Analytics		04/06/2024
Northern Africa	Cadi Ayyad University (Morocco)	Morocco	Bachelor's Degree In Fundamental Studies: Mathematics And Computer Sciences			Https://Www.Uca.Ma/Fr	04/06/2024
Northern Africa	Cadi Ayyad University (Morocco)	Morocco	BSc In Artificial Intelligence And Robotization			Https://Www.Uca.Ma/Fr	04/06/2024
Northern Africa	Cadi Ayyad University (Morocco)	Morocco	BSc Computer Science			Https://Www.Uca.Ma/Fr	04/06/2024
Northern Africa	Cairo University	Egypt	MSc In Big Data Analytics			Https://Cu.Edu.Eg/Faculties	04/06/2024
Northern Africa	Cairo University	Egypt	Masters In Computer And Informatics Sciences Applications			Https://Cu.Edu.Eg/Faculties	04/06/2024
Northern Africa	Cairo University	Egypt	PhD In Computer And Informatics Sciences Applications			Https://Cu.Edu.Eg/Faculties	04/06/2024
Northern Africa	Canadian International College (Cic)	Egypt	BSc Data Science		Https://Www.Cic-Cairo.Edu.Eg/Major-Of-Data-Science/		04/06/2024

Northern Africa	German University In Cairo (Guc)	Egypt	MSc In Computer Science And Engineering		Https://Www.Guc.Edu.Eg/En/Academic_Programs/Postgraduate_Studies/Master_Programs		04/06/2024
Northern Africa	Hassan Ii University (Morocco)	Morocco	MSc In Data Science And Big Data			Https://Www.Univh2c.Ma/	04/06/2024
Northern Africa	Jamhuriya University Of Science And Technology (Egypt)	Egypt	BSc In Computer Applications	Computer & Information Technology	Https://Cit.Just.Edu.So/Departments/Bachelor-Of-Science-In-Computer-Applications		04/06/2024
Northern Africa	Manouba University (Tunisia)	Tunisia	Master Of Research In Computer Science		Http://Www.Uma.Rnu.Tn/Page.Php?Code=45		05/06/2024
Northern Africa	Mohammed V University (Morocco)	Morocco	MSc In Artificial Intelligence			Http://Www.Um5.Ac.Ma/Um5/	05/06/2024
Northern Africa	The American University In Cairo (Egypt)	Egypt	BSc Data Science		Https://Www.Aucegypt.Edu/Academics/Undergraduate-Programs/Data-Science		04/06/2024
Northern Africa	Tunis University (Tunisia)	Tunisia	Licence In Computer Science	Mathematical, Physical And Natural Sciences	Http://Www.Utm.Rnu.Tn/Utm/Fr/Formation--Mastere		05/06/2024
Northern Africa	Tunis University (Tunisia)	Tunisia	Masters In Computer Science	Mathematical, Physical And Natural Sciences	Http://Www.Utm.Rnu.Tn/Utm/Fr/Formation--Mastere		05/06/2024
Northern Africa	Tunis University (Tunisia)	Tunisia	PhD In Computer Science	Mathematical, Physical And Natural Sciences	Http://Www.Utm.Rnu.Tn/Utm/Fr/Formation--Doctorat		05/06/2024
Northern Africa	University Of Sfax (Tunisia)	Tunisia	Research Master In Computer Science (Si)		Https://Isimsf.Rnu.Tn/Fra/S174/Pages/89/Mastere-De-Recherche-Sciences-Informatique-(Si)		05/06/2024
Northern Africa	University Of Sfax (Tunisia)	Tunisia	MSc In Data Science		Https://Isimsf.Rnu.Tn/Fra/S174/Pages/91/Maste		05/06/2024

					re-De-Recherche-Datascience-(Ds)		
Island Nations	University Of Mauritius	Mauritius	BSc (Hons) In Data Science	Information, Communication And Digital Technologies	Https://Www.Uom.Ac.Mu/Foicdt/Index.Php/Programmes/Undergraduate		05/06/2024
Island Nations	University Of Mauritius	Mauritius	MSc Artificial Intelligence	Information, Communication And Digital Technologies	Https://Www.Uom.Ac.Mu/Foicdt/Index.Php/Programmes/Postgraduate		05/06/2024
